# Discovery of Novel Hydroxyimine-Tethered Benzenesulfonamides as Potential Human Carbonic Anhydrase IX/XII Inhibitors

**DOI:** 10.1021/acsmedchemlett.3c00094

**Published:** 2023-05-08

**Authors:** Mudasir Nabi Peerzada, Daniela Vullo, Niccolò Paoletti, Alessandro Bonardi, Paola Gratteri, Claudiu T. Supuran, Amir Azam

**Affiliations:** † Medicinal Chemistry and Drug Discovery Research Laboratory, Department of Chemistry, Jamia Millia Islamia, Jamia Nagar, New Delhi-110025, India; ‡ Department of NEUROFARBA, Section of Pharmaceutical and Nutraceutical Sciences, Laboratory of Molecular Modeling, Cheminformatics & QSAR, University of Florence, Polo Scientifico, Via U. Schiff 6, 50019 Sesto Fiorentino, Florence, Italy

**Keywords:** benzenesulfonamides, carbonic anhydrases, SLC-0111, anticancer, drug discovery

## Abstract

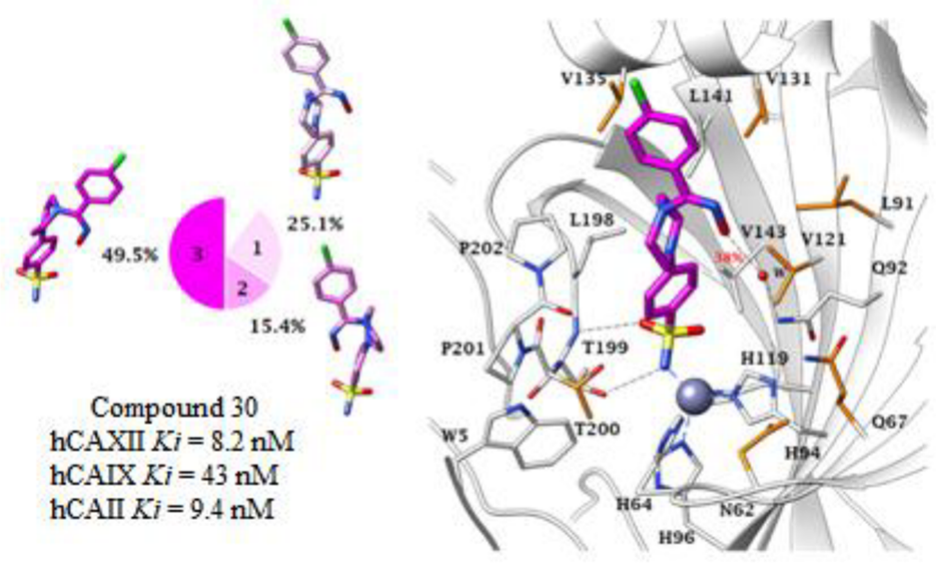

To discover novel carbonic anhydrase (CA, EC 4.2.1.1) inhibitors for cancer treatment, a series of 4-{4-[(hydroxyimino)methyl]piperazin-1-yl}benzenesulfonamides were designed and synthesized using SLC-0111 as the lead molecule. The developed novel compounds **27**–**34** were investigated for the inhibition of human (h) isoforms hCA I, hCA II, hCA IX, and hCA XII. The hCA I was inhibited by compound **29** with a *K*_i_ value of 3.0 nM, whereas hCA II was inhibited by compound **32** with a *K*_i_ value of 4.4 nM. The tumor-associated hCA IX isoform was inhibited by compound **30** effectively with an *K*_i_ value of 43 nM, whereas the activity of another cancer-related isoform, hCA XII, was significantly inhibited by **29** and **31** with a *K*_i_ value of 5 nM. Molecular modeling showed that drug molecule **30** participates in significant hydrophobic and hydrogen bond interactions with the active site of the investigated hCAs and binds to zinc through the deprotonated sulfonamide group.

Carbonic anhydrases (CAs, EC 4.2.1.1) are ubiquitous metalloenzymes that are involved in the reversible catalytic hydration of carbon dioxide in cells to HCO_3_^–^ and H^+^. The human carbonic anhydrases (hCAs) pertain to the α-family of CAs and are associated with several pathophysiological processes, such as electrolyte secretion, diverse biosynthetic reactions, pH regulation, CO_2_ homeostasis, and tumorigenesis.^1,2^ Fifteen isoforms of this class of enzymes have been discovered in humans, and they have distinct structure, function, and kinetic properties, localization, and catalytic behavior.^3^ The overexpression of CAs is associated with a broad spectrum of diseases, including glaucoma, neuropathic pain, edema, obesity, and tumors. Therefore, at present CA inhibitors are used for treatment of epilepsy, edema, glaucoma, obesity, and several cancers.^4^ Although scientists have discovered various hCA isoforms as potential and validated therapeutic targets for various diseases, further research is in progress to find the involvement of the CAs with other pathological disorders.^5^

The CA isoforms hCA IX, hCA XII, and hCA II are involved in several metabolic processes and pH regulation in hypoxic tumors in which, due to the insufficient availability of molecular oxygen, the oxidative phosphorylation process of glucose is reduced and leads to a decrease in ATP production. Under the conditions when there is a lack of ATP production in the tricarboxylic acid cycle due to hypoxia, the cells meet the ATP production by adopting the alternative glycolytic pathway, in which lactic acid is produced as one of the bioproducts.^6^ Under normal conditions hCA IX is expressed in some cells, but it gets overexpressed in several cancers, such as colorectal, breast, brain, etc., in hypoxia due to sturdy transcriptional activation induced by hypoxia-inducible factor HIF-1.^7^ The overexpression of hCA IX in hypoxic tumors causes a decrease in the pH of the extracellular matrix and thereby enhances the survival and progression of cancer. More importantly, hCA IX overexpression enhances the chemoresistance of anticancer drugs that are weakly alkaline in nature.^8^ Consequently, hCA IX is one of the attractive targets for the design and development of anticancer drugs for both early-stage and metastatic hypoxic cancers.^9^ The effect of hCA XII overexpression is mediated by hypoxia and estrogen receptors in hypoxic tumors. It has been documented that hCA XII expression is up-regulated under hypoxic conditions like that of the hCA IX isoform.^10^ hCA XII is the cancer-associated isoform and is overexpressed in many forms of human cancer, including renal, pancreatic, gut, oral, brain, lung, and ovarian cancers. Therefore, hCA XII is also one of the remarkable biomarkers for the inhibition of various hypoxic tumors at primary and metastases stages.^11^

As the CAs are zinc metalloenzymes, the active site contains Zn^2+^ that is tetrahedrally coordinated to three histidine amino acid residues and one water molecule/hydroxide ion. The nitrogen atom of the imidazole ring of these residues is linked to the Zn^2+^ in the active-site region, and this site is present at the bottom half of the hydrophobic and hydrophilic cleft. Compounds of medicinal value can inhibit the functioning of CAs by interfering with this active site and avert the associated function.^12^ Ostensibly, several primary sulfonamides have been used for the treatment of glaucoma and epilepsy and as diuretics for decades, and this class of compounds is most investigated as carbonic anhydrase inhibitors (CAIs).^13^ For example, acetazolamide (AAZ) and dorzolamide are primary sulfonamides and are classified as first- and second-generation drugs used as CAIs.^14^ In addition, there are several compounds containing the SO_2_NH_2_ group that have shown significant inhibition of CAs, including brinzolamide, ethoxzolamide, indapamide, and celecoxib^3^ (Figure 1).

**Figure 1:**
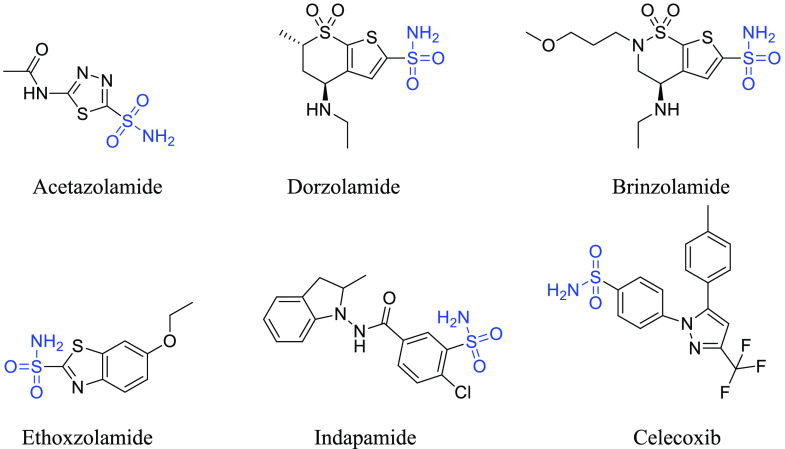
Some potent carbonic anhydrase inhibitors containing the SO_2_NH_2_ zinc-binding group.

Author: The benzenesulfonamide scaffold is widely used in the development of CAIs. This fragment is a significant zinc-binding group (ZBG) and binds to the zinc central metal atom of the CA through a coordinate bond to cause the inhibition.^15^ Several benzenesulfonamide derivatives were synthesized by various medicinal chemists to develop potent and selective hCA inhibitors.^16,17^ One of such potent inhibitors is the 4-(4-fluorophenylureido)benzenesulfonamide (SLC-0111) that is in phase II clinical trials for the treatment of solid hypoxic metastatic tumors.^18,19^ Therefore, in the present investigation, SLC-0111 was taken as the lead compound to develop a novel series of CAIs. Molecules containing the -NOH group, such as psammaplin C, have recently been reported as potent inhibitors of CAIs.^20,21^ Psammaplin C, a natural product, has shown significant inhibition of various CA isoforms, with *K*_i_ values in the nanomolar range, and contains an -NOH group in addition to an SO_2_NH_2_ group. Psammaplin C was investigated against the panel of hCA isoforms, and it inhibited hCA I, hCA II, hCA IX, and hCA XII with *K*_i_ values of 48.1 nM, 88.0 nM, 12.3 nM, and 0.79 nM, respectively.^21^

Piperazine heterocycles are widely used in anticancer drug development, and recently several compounds having this ring have shown potent carbonic anhydrase inhibitory activity.^22,23^ Scaffold hopping is one of the modern strategies for the discovery of novel drug candidates and is used in the invention of isofunctional chemotypes with molecular structures significantly distinct from those of lead molecules.^24,25^ Inspired by this strategy, in the present research work, a novel series of small-molecule 4-{4-[(hydroxyimino)methyl]piperazin-1-yl}benzenesulfonamides were developed by combining the benzenesulfonamide core of SLC-0111 with the >C=NOH group present in Psammaplin C, linked through the significantly bioactive piperazine heterocyclic scaffold (Figure 2).

**Figure 2:**
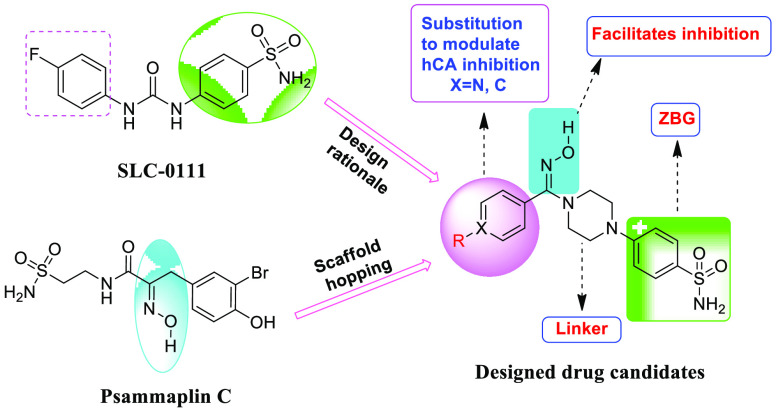
Drug design strategy of the synthesized carbonic anhydrase inhibitors.

The synthetic strategy employed for the synthesis of target compounds is depicted in Scheme 1. The intermediates **9**–**16** were obtained by reacting various aldehydes (**1**–**8**) with hydroxylamine under reflux in a basic medium.^26^ The carboximidoyl chlorides **17**–**24** were prepared by chlorinating the intermediates **9**–**16** with *N-*chlorosuccinimide.^27^ The 4-fluorobenzenesulfonamide **25** was reacted with piperazine under reflux to obtain the compound **26**. In the next step the carboximidoyl chlorides **17**–**24** were reacted with compound **26** to get the target hydroxyimine-derived benzenesulfonamides **27**–**34**. The structures of all the target compounds were established with the help of ^13^C NMR, ^1^H NMR, and ESI-MS, and the purity was elucidated by CHNS elemental analysis. ^1^H NMR showed that the -OH group resonates in the range of δ 10.54 to 9.42 ppm as a singlet in compounds **24**–**34**. However, the singlet obtained in the range of δ 163.41 to 152.98 ppm in the ^13^C NMR of compounds **24**–**34** corresponds to carbon atom of the >C=N- group. The two hydrogen atoms of the >SO_2_NH_2_ group showed signals in the range of δ 7.09 to 7.03 ppm in the ^1^H NMR for compounds **24**–**34**. All the hydrogen atoms of the piperazine ring showed signals in the aliphatic region of the ^1^H NMR spectra of the target compounds. Similarly, the signals obtained in the aliphatic region of the ^13^C NMR correspond to the carbon atoms of the piperazine heterocycle.

**Scheme 1:**
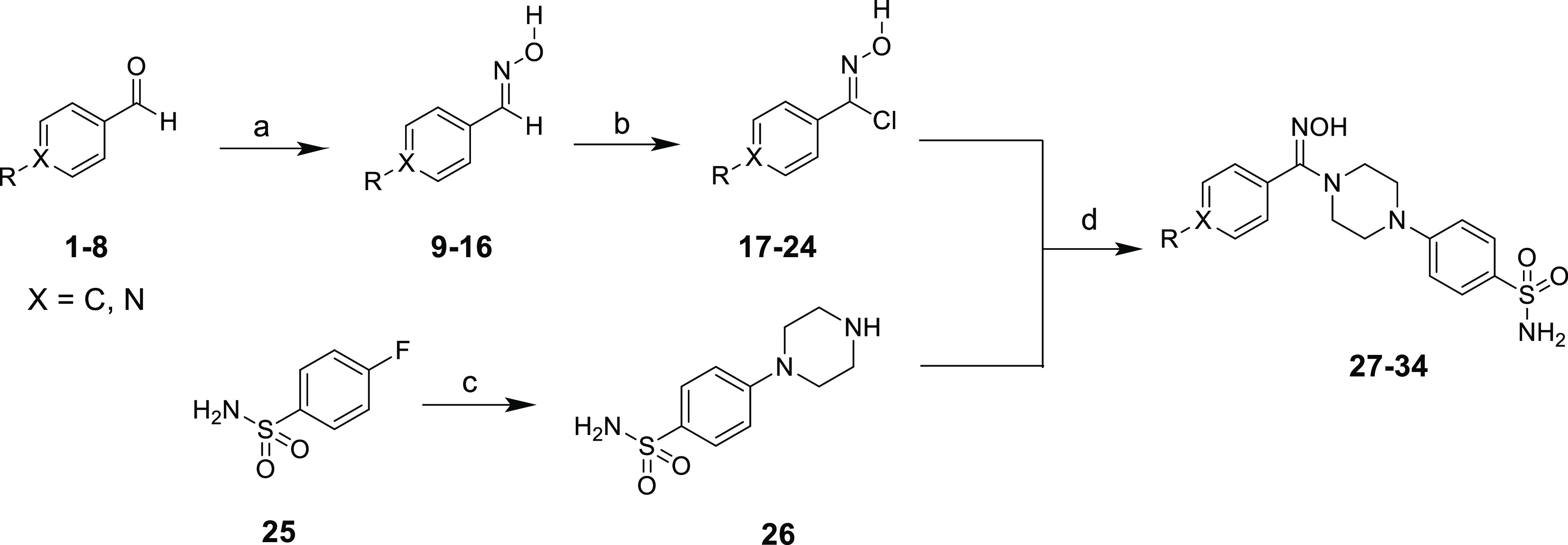
Strategy Employed for the Synthesis of Target Compounds **Reagents and conditions:** (a) C_2_H_5_OH, NH_4_OH, NaOH, reflux, 10–15 h; (b) DMF, NCS, 60 °C, 5–7 h. (c) piperazine, H_2_O, 100 °C, overnight; (d) Na_2_CO_3_, 50% THF:H_2_O, room temperature, 24–48 h.

The hCA inhibition profiles of benzenesulfonamides **27–34** synthesized in this study were investigated by stopped-flow CO_2_ hydrase assay and AAZ was taken as the reference drug.^28^ The compounds were screened against a panel of hCA isoforms including hCA I, hCA II, hCA IX, and hCA XII. Cytosolic hCA II and transmembrane hCA XII isoforms are upregulated in glaucoma and are therefore prominent targets for intraocular pressure-maintaining drugs.^28−30^ Isoforms hCA IX and hCA XII are overexpressed in cancer patients and are therefore validated targets for cancer treatment.^31^ In contrast, the cytosolic hCA I isoform is off target and is inhibited by sulfonamides effectively.^32^ The assessed results for the inhibition of screened isoforms and the selectivity index (SI) values of designed drug molecules **27**–**34** compared with the standard AAZ are mentioned in Tables 1 and 2.

**Table 1:** Inhibition Profile of Human CA Isoforms hCA I, hCA II, hCA IX, and hCA XII for Compounds **27–34** Using Acetazolamide (AAZ) as a Reference Drug

			***K*_i_ (nM)**^a^			
**Compd**	**X**	**R**	**hCA I**	**hCA II**	**hCA IX**	**hCA XII**
**27**	N	–	35.0	5.0	110.0	52.3
**28**	C	OH	241.7	80.7	1179.4	7.7
**29**	C	H	3.0	9.0	121.0	5.5
**30**	C	Cl	3.5	9.4	43.0	8.2
**31**	C	OCH_3_	4.0	7.6	739.1	5.5
**32**	C	NO_2_	4.1	4.4	911.5	>10000
**33**	C	F	80.7	6.6	1339.0	106.2
**34**	C	CH_3_	4.5	6.0	>10000	93.4
**AAZ**	–	–	250	12.1	25.7	5.7

a Mean taken from 3 different assays (the errors were in the range of ±5–10% of the reported values).

**Table 2:** Selectivity Index (SI) for Compounds **27–34** and AAZ Calculated as a Ratio between *K*_i_ hCA I and *K*_i_ hCA II

	**SI**			
**Compd**	**hCA IX/I**	**hCA IX/II**	**hCA XII/I**	**hCA XII/II**
**27**	3.1	22.0	1.5	10.5
**28**	4.9	14.6	0.03	0.1
**29**	40.3	13.4	1.8	0.6
**30**	12.3	4.6	2.3	0.9
**31**	184.8	97.3	1.4	0.7
**32**	222.3	207.2	>2439.1	>2272.7
**33**	16.6	202.9	1.3	16.1
**34**	>2222.2	>1666.7	20.8	15.6
**AAZ**	0.1	2.1	0.02	0.5

To determine the structure–activity relationship (SAR) trends, the CA inhibitory activities of the investigated series of compounds were deeply analyzed. It was found that all the compounds of the series inhibited the activity of hCA I in the nanomolar range. Compound **29**, having an unsubstituted phenyl ring attached to the >C=NOH group, showed an inhibition constant *K*_i_ value of 3.0 nM for the hCA I isoform, and substitution of a chlorine atom at the *para* position of this phenyl ring in compound **30** led to the molecule having a *K*_i_ value of 3.5 nM for the inhibition of hCA I. Replacement of the -Cl substituent with a -OCH_3_ group in compound **31** led to the discovery of a chemotype that inhibited the hCA I activity with a *K*_i_ value of 4.0 nM. Incorporation of an electron-withdrawing group, -NO_2_, in compound **32** led to a novel compound having a *K*_i_ value of 4.1 nM. However, the compound having a -CH_3_ substituent at the *para* position showed the inhibition of hCA I with a *K*_i_ value of 45 nM. Introduction of a pyridine ring instead of a phenyl ring in compound **27** caused a decrease in potency, and the observed *K*_i_ value for hCA I was 35.0 nM. However, the introduction of -F and -OH groups in compounds **28** and **33** caused sharp declines in potency. Although all the compounds of the series appeared to be more potent than AAZ, compound **29** showed higher potency and the compound having the hydroxyl group showed the minimum potency. The overall potency followed the pattern as H > Cl ≫ OMe > NO_2_ > Me > Py > F > OH, and the SAR is substituent dependent for the hCA I isoform.

Further, all the compounds of this series inhibited hCA II in the 4.4 to 80.7 nM range. Compound **32**, containing the NO_2_ group, appeared to be a potent inhibitor of this screened isoform, and the inhibition constant *K*_i_ was observed to be 4 nM. The compound containing a pyridine ring showed significant inhibition of hCA II, and the recorded *K*_i_ value was found to be 5.0 nM. However, compound **34**, containing the -CH_3_ substituent at the *para* position of the aryl ring linked directly with >C=NOH, showed an inhibition constant *K*_i_ value of 6.0 nM for hCA II, while compound **33** containing the F substituent showed an inhibition constant of 6.6 nM. Incorporation of a -OCH_3_ substituent led to the discovery of a compound that showed an inhibition constant of 7.6 nM. Compound **29** containing the unsubstituted phenyl ring showed a *K*_i_ value of 9.0 nM, while the introduction of a chlorine atom at the *para* position of the aryl ring led to compound **30**, having a *K*_i_ value of 9.4 nM for hCA II. Compound **28**, having the -OH group, appeared to be least active for the inhibition of the hCA II isoform; its observed *K*_i_ value was 80.7 nM. The overall pattern of the inhibition of hCA II is NO_2_ > Py > -CH_3_ > F > OCH_3_ > phenyl > Cl > OH.

In the case of the hCA IX isoform, compound **30** having a -Cl group at the *para* position of the ring adjacent to >C=NOH, showed the significant inhibition and an observed *K*_i_ value of 43 nM. The pyridine derivative **27** inhibited the hCA IX activity with an inhibitor constant value of 110 nM. However, compound **29**, having a phenyl group linked to >C=NOH, inhibited the hCA IX activity with a *K*_i_ value of 121 nM. Compound **31**, having a -OCH_3_ group, inhibited hCA IX with a *K*_i_ value of 739.1 nM. Compound **32**, containing an electron-withdrawing group, inhibited the activity of hCA IX with a *K*_i_ value of 911.5 nM, and compound **28**, having an -OH group, inhibited the activity of this isoform with a *K*_i_ value of 1179.4 nM, while compound **33**, having a fluoro substituent, inhibited the activity of hCA IX with a *K*_i_ value of 1339.0 nM. Compound **34**, containing a methyl substituent, appeared to be least active for the inhibition of hCA IX, with an observed *K*_i_ value of >10000 nM. The pattern of inhibition is Cl > Py > phenyl > OCH_3_ > NO_2_ > OH > F > CH_3_ for hCA IX.

All the compounds of the present series inhibited hCA XII significantly, and most of these compounds inhibited the activity of this tumor-associated enzyme in the nanomolar range. Compounds **29** and **31**, having phenyl and -OCH_3_ substituents directly linked to the >C=NOH core, inhibited the hCA XII activity in the nanomolar range, the observed *K*_i_ value for both these compounds being 5.5 nM. Compound **28**, having an -OH substituent at the *para* position of the ring adjacent to >C=NOH, inhibited the hCA XII activity with a *K*_i_ value of 7.7 nM. Similarly, compound **30**, having a chlorine atom at the *para* position of the ring, inhibited hCA XII with a *K*_i_ value of 8.2 nM. The pyridine derivative **27** inhibited the activity of hCA XII with an inhibition constant value of 52.3 nM, whereas compound **34**, having a -CH_3_ group, inhibited the activity of hCA XII with a *K*_i_ value of 93.4 nM. Compound **33**, having a fluoro atom at the *para* position of the aryl ring, inhibited the hCA XII activity with an inhibition constant value of 106.2 nM. However, compound **32**, having a NO_2_ group at the *para* position of the aryl ring adjacent to >C=NOH, inhibited the hCA XII activity weakly, and the *K*_i_ value was >10000 nM. The overall activity pattern is phenyl = OMe > OH > Cl > py > CH_3_ > F > NO_2_. These SAR trends can be determined from the data given in Table 1.

In order to rationalize how compound **30**, the most potent of the synthesized series, interacts with the human off-target carbonic anhydrase I and II isozymes and the tumor-related isoforms IX and XII, a computational procedure was applied based on docking and molecular dynamics (MD) simulations carried out on both isomers (*Z*) and (*E*). According to the literature,^33−36^ all docking solutions provided poses with the benzenesulfonamide group deeply bound to the zinc ion through the deprotonated nitrogen atom (SO_2_NH^–^). Stabilization of the ligand within the active site was also supported by hydrophobic contacts of the aromatic ring with residues L198, V121 (A121 in CA I isoform), V143, and W205 and by the formation of two H-bonds between the sulfonamide NH^–^ and S=O with the side chain OH and backbone NH of T199, respectively.

Inspection of the trajectories from 100 ns long MD simulations pointed out the presence of up to three preferential conformations of the compound in each of the four different isoforms of the enzyme (Figures 3 and 4). In order to take into account the time-dependent adaptation process of the ligand in the CA’s active site, the dynamic behavior of both isomers of ligand **30** was analyzed without considering the first 10 ns of the MD trajectories (black dashed line in Figures 3 and 4). As expected, the coordination bond Zn–N and the two H-bonds engaged between the sulfonamide moiety and T199 are stably maintained for the entire course of the MDs. Ligand motions within the binding clefts reflect the features of each isoform’s active sites. Within the smallest binding site of CA I, (*Z*)-**30** and (*E*)-**30** assume two and three conformations, respectively. The most stable conformer of the (*Z*) isomer is maintained for about 66% of the entire dynamic duration, also thanks to the persistence (83%) of the interaction between the hydroxamic group and Q92 to which the group binds via a water-bridge bond (Figure 5A). In the same active site the (*E*) isomer undergoes rapid interconversion between two main conformers having the -NOH group oriented toward the exit of the active site, in water-bridged H-bond distance (17%) with the side chain NH_2_ of Q92 (Figure 6A).

**Figure 3:**
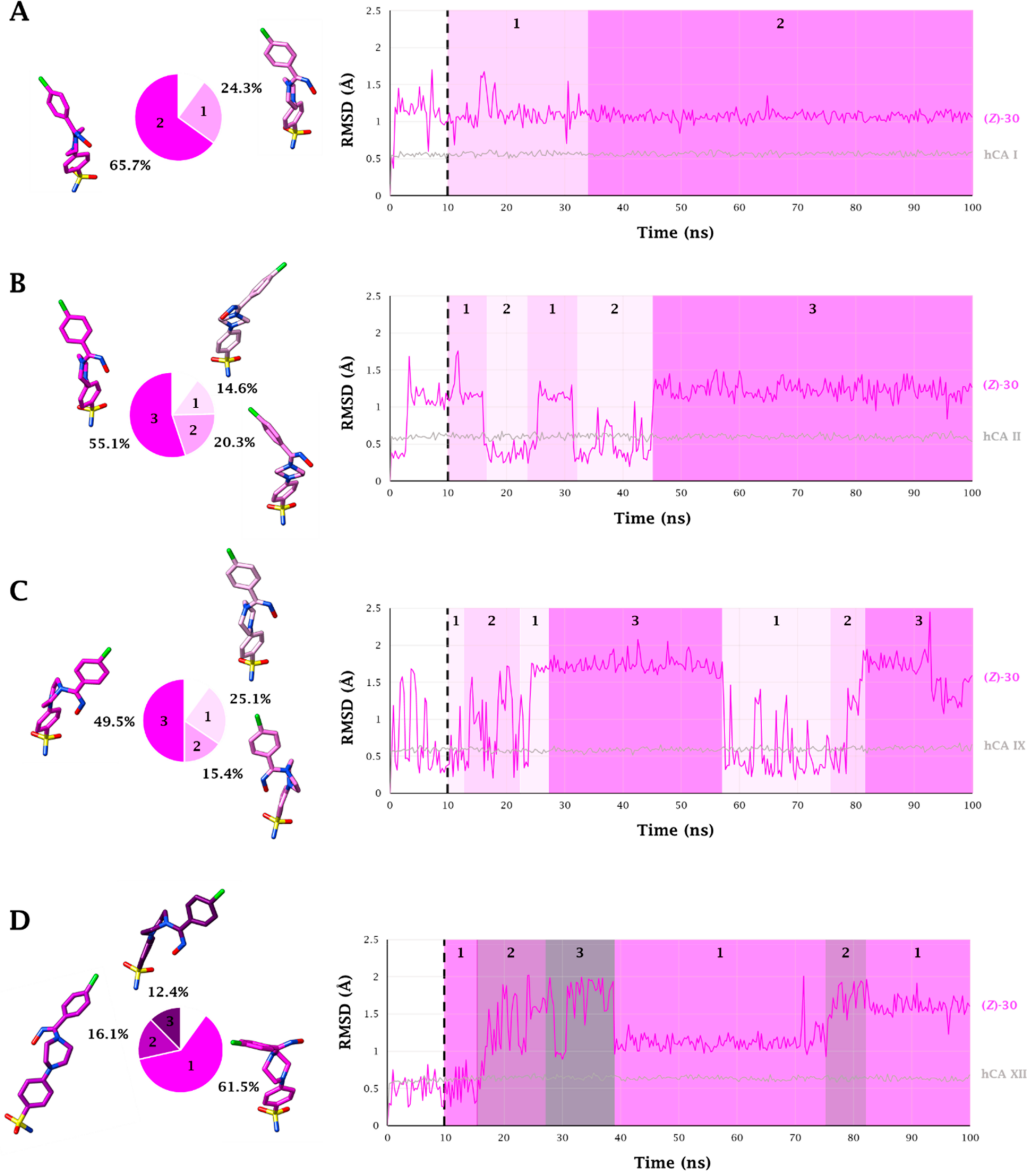
100 ns long MD trajectory of (Z)-**30** within A) hCA I, B) hCA II, C) hCA IX, and D) hCA XII active sites. Pie representations of the representative conformers per cluster are depicted on the left.

**Figure 4:**
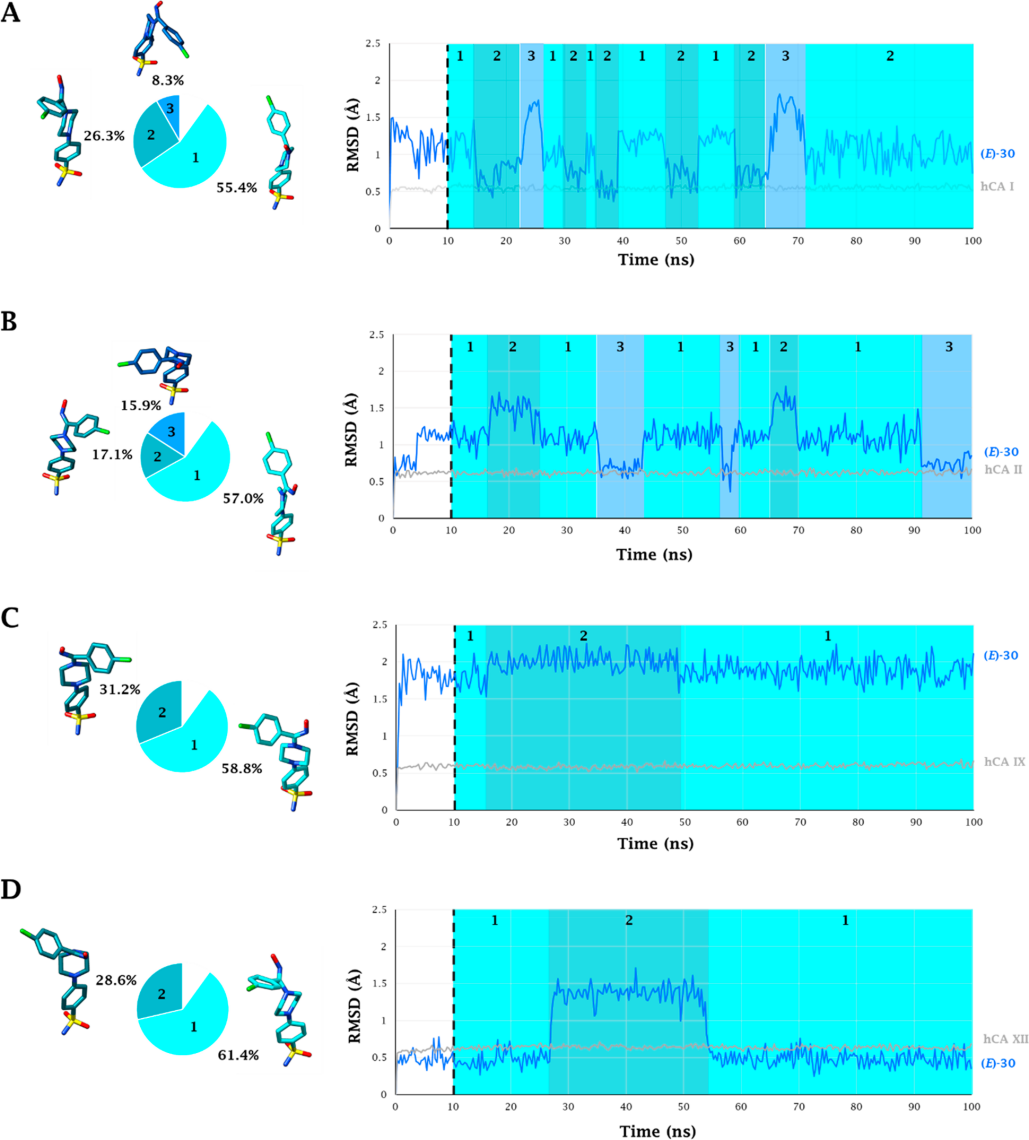
100 ns long MD trajectory of (*E*)-**30** within A) hCA I, B) hCA II, C) hCA IX, and D) hCA XII active sites. Pie representations of the representative conformers per cluster are depicted on the left.

**Figure 5:**
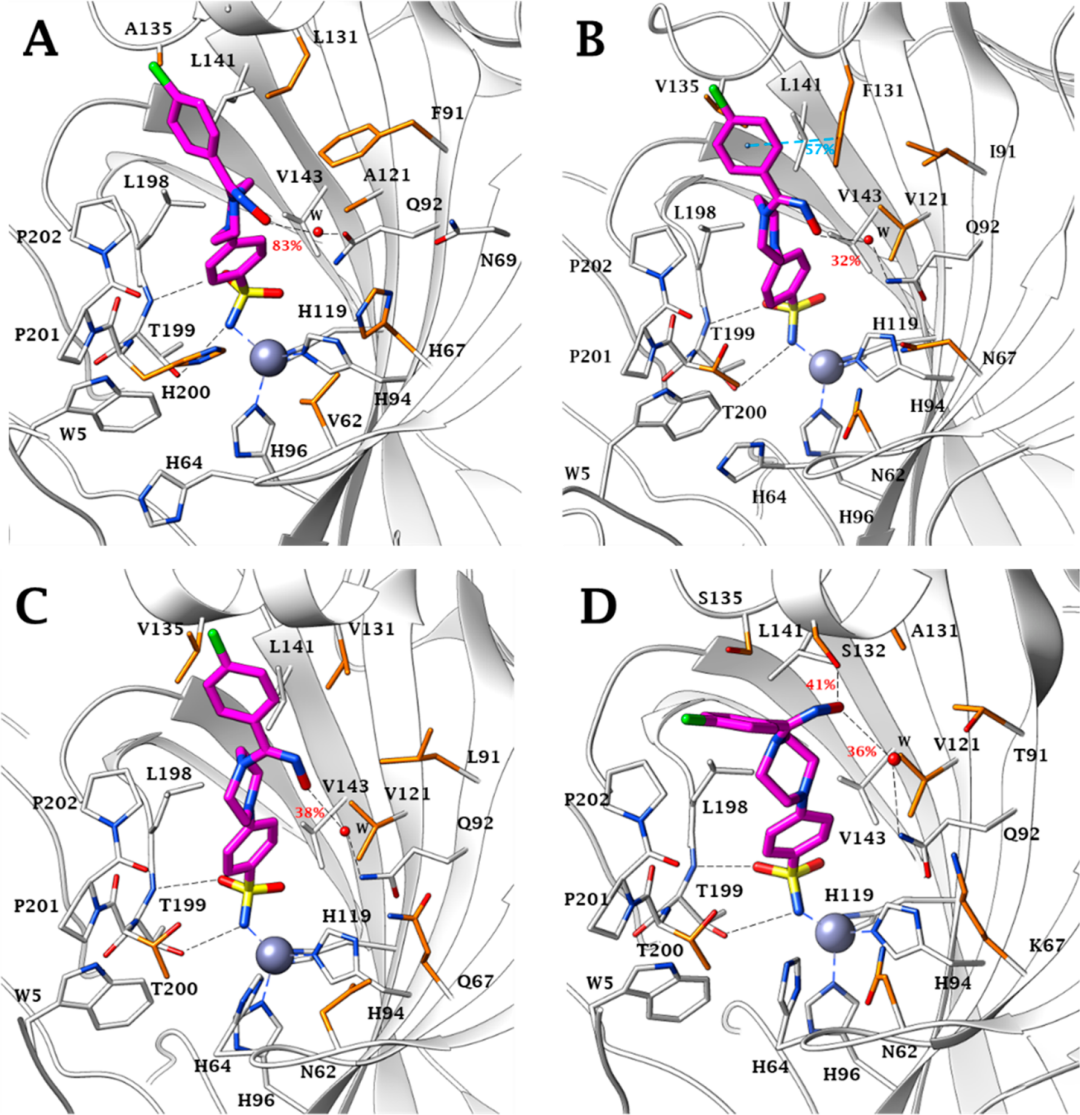
MD most persistent conformers of (*Z*)-**30** (magenta) into A) hCA I (65.7% persistence), B) hCA II (55.1% persistence), C) hCA IX (49.5% persistence), and D) hCA XII (61.5% persistence) active site. Water molecules are shown as red spheres, while H-bonds are shown as black dashed lines and the π–π stacking interactions are shown as cyan dashed lines. Dashed bond occupancy over the MD simulation is indicated as a percentage. The 100% occupancy of the binding to the zinc ion and with T199 is omitted. Mutated residues are in orange.

**Figure 6:**
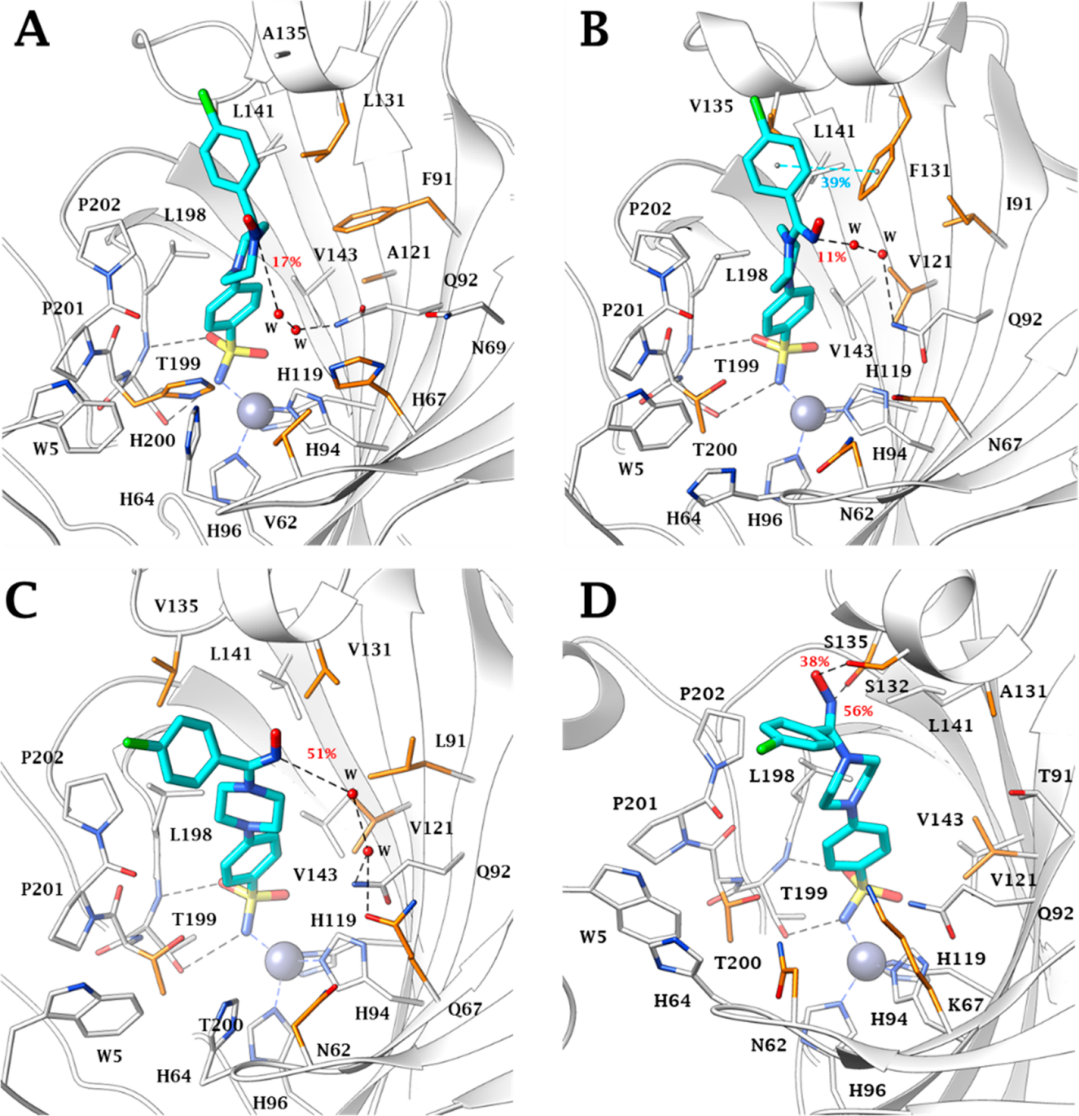
MD most persistent conformers of (*E*)-**30** (cyan) into A) hCA I (55.4% persistence), B) hCA II (57.0% persistence), C) hCA IX (58.8% persistence), and D) hCA XII (61.4% persistence) active site. Water molecules are shown as red spheres, while H-bonds are shown as black dashed lines and the π–π stacking interactions are shown as cyan dashed lines. Dashed bond occupancy over the MD simulation is indicated as percentage. The 100% occupancy of the binding to the zinc ion and with T199 is omitted. Mutated residues are in orange.

The first half of the MD simulation of (*Z*)-**30** within CA II is characterized by the frequent switching between two conformations (1 and 2 in Figure 3B). In the second half of the MD the ligand stabilizes in a third conformation due to both the water-bridged H-bond formed between the hydroxamic group and Q92 and the π–π stacking contacts of the 4-chlorobenzene portion and the aromatic ring of F131 (Figure 5B). It is the interaction with this latter residue, peculiar to the CA II isoenzyme, that leads the 4-chlorobenzene moiety toward the lipophilic area of the receptor, increasing the lipophilic interactions with the residues V135, L141, P201, and P202 (Figure 5B). The hydroxamic nitrogen atom of the most represented conformation in the (*E*) isomer (57%) engages a water-bridged H-bond with the Q92 (11%) and π–π stacking interactions with the peculiar F131 (39%) (Figure 6B). Nevertheless, the orientation of this group toward the entrance of the active site can cause the conformational fluctuation observed in the MD trajectory (Figure 4B).

The F131/V131 (CA II/CA IX) and F91/I91 (CA I/CA IX) mutations make the binding area of the CA IX roomier, thus allowing (*Z*)-**30** and (*E*)-**30** to move freely and switching between three and two main conformations, respectively. In all those the 4-chlorobenzene ring undergoes hydrophobic interactions with the lipophilic cleft lined by the residues V131, V153, P201, and P202 (Figures 3–6C). The about 50% represented (*Z*)-**30** conformation is involved in a water-bridged H-bond between the hydroxyimine OH and the side chain NH_2_ of Q92 (38%), while the conformer (*E*)-**30** lodges the nitrogen atom of the -NOH group in water-bridged H-bond distance with the NH_2_ side chains of Q92 and Q67 (51%) (Figure 6C).

The prevalent (about 62%) conformation of (*Z*)-**30** (1, Figure 3D) at the CA XII binding site places the oxygen atom of the hydroxamic moiety in H-bond distance with S132, a peculiar amino acid residue of CA XII (41% persistence) (Figure 5D), leading to a slightly different orientation of the ligand than observed in the active sites of the other isoenzymes. The anchorage of the hydroxamic group to the enzyme is reinforced by a water-bridged H-bond with Q92 (36% persistence) (Figure 5D). The most persistent conformation of (*E*)-**30** (1, Figure 4D), is stabilized by H-bonds engaged by the -NOH group with both the side-chain OH of the peculiar S132 and S135 (38% and 56% persistence, respectively) (Figure 6D).

The analysis of the binding modes of both the (*Z*) and (*E*) isomers of ligand **30** within the four carbonic anhydrase isoforms provides convincing support to the experimentally detected enzymatic inhibition data motivating the best inhibitory profile toward the isoforms I, II, and XII of the enzyme within which the compound is held more stably by hydrophobic and H-bond interactions.

Next, we examined the drug-likeness properties and other physicochemical properties of the synthesized compounds **27**–**34** using SwissADME software^37,38^ The Lipinski rule is considered as one of the significant rules in determining the theoretical pharmacological behavior of drug candidates and is extensively applied in drug discovery. The Lipinski rule is an important rule for assessing the pharmacological activity of drug candidates prior to *in vivo* investigation and is therefore extensively employed in drug design and development. The biologically active molecule must implement certain parameters to be potentially used as a drug candidate for patient treatment. These parameters include (1) molar mass < 500, (2) number of hydrogen bond acceptors < 10, (3) total number of hydrogen bond donors < 5, and (4) MlogP < 5.^39^ Compounds **27**–**34** complied with the Lipinski rule and exhibited considerable oral bioavailability scores, exerting appreciable pharmacokinetic profiles and significant drug-likeness properties. Moreover, the predictive results manifested that these compounds could not penetrate blood–brain barrier (BBB). All the compounds showed bioavailability scores the same as that of AAZ which determines the desirable bioavailability of the compounds and enviable pharmacokinetic properties. In addition, compounds **27**–**34** follow the Lipinski rule for the hydrogen bond donors and acceptors that demonstrate the drug-likeness properties of these screened compounds. The physicochemical properties, lipophilicity, and other drug-likeness properties of the target compounds **27**–**34** are indicated in Table 3.

**Table 3:** Drug-Likeness Properties of Compounds **27–34** and Acetazolamide

**Compd**	**MW** (g/mol)	**MlogP < 5**	**Hydrogen bond acceptors**	**Hydrogen bond donors**	**Lipinski violations**	**Bioavailability score**	**BBB permeant**	**PAINS**
**27**	361.42	0.57	6	2	0	0.55	No	0
**28**	376.43	1.08	6	3	0	0.55	No	0
**29**	360.43	1.60	5	2	0	0.55	No	0
**30**	394.88	2.11	5	2	0	0.55	No	0
**31**	390.46	1.62	6	2	0	0.55	No	0
**32**	405.43	1.52	7	2	0	0.55	No	0
**33**	378.42	1.99	6	2	0	055	No	0
**34**	374.46	1.84	5	2	0	055	No	0
**AAZ**	221.24	–2.34	6	2	0	0.55	0	0

In conclusion, this study led to the discovery of small-molecule 4-{4-[(hydroxyimino)methyl]piperazin-1-yl}benzenesulfonamides as a novel class of compounds that showed significant CA inhibitory activity and could be used as anticancer and antiglaucoma agents in the future. All developed inhibitors **27**–**34** bind to the zinc ion of the hCA through the -NH^–^ group of >SO_2_, as all other primary sulfonamides and as validated by molecular docking studies. Compound **30** emerged as one drug candidate that inhibits the activity of tumor-associated hCA IX and hCA XII with inhibition constant *K*_i_ values of 43 nM and 8.2 nM, demonstrating the potential anticancer activity of the chemotype. A substantial number compounds of the screened series in this study inhibited the activity of the human carbonic anhydrases hCA I, hCA II, hCA IX, and hCA XII at nanomolar concentrations, demonstrating that such compounds could be used as potential hCA inhibitors and treated as lead candidates. The results assessed for the pharmacological effect demonstrated that such small-molecule benzenesulfonamides could be presumed as drug candidates for the treatment of multiple malignancies through tumor-associated CA inhibition.

## Supporting Information Available

Synthesis procedure for intermediates **9**–**24** and **26**, and target compounds **27**–**34**; characterization data; biological and bioinformatics protocols; and ^1^H and ^13^C NMR of compounds **27**–**34** (PDF)

## Abbreviations

AAZ: acetazolamide
hCA: human carbonic anhydrase
CA: carbonic anhydrase
CAI: carbonic anhydrase inhibitor
ZBG: zinc-binding group
HIF-1: hypoxia-inducible factor-1
MD: molecular dynamics
