# Supplementary material for: Discovery of Novel Hydroxyimine-Tethered Benzenesulfonamides as Potential Human Carbonic Anhydrase IX/XII Inhibitors
Source: ACS Med Chem Lett. 2023 May 8;14(6):810–9. doi: 10.1021/acsmedchemlett.3c00094 (PMC10258898; doi:10.1021/acsmedchemlett.3c00094)
Supplement: Supplementary file 1 — ml3c00094_si_001.pdf [file ml3c00094_si_001.pdf]

Supporting Information

Discovery of Novel Hydroxyimine Tethered Benzenesulfonamides as Potential Human Carbonic Anhydrase IX/XII Inhibitors

Mudasir Nabi Peerzada[1], Daniela Vullo[2], Niccolò Paoletti[2], Alessandro Bonardi[2], Paola Gratteri[2], Claudiu T. Supuran[2,*], Amir Azam[1,*]

[1]*Medicinal Chemistry Research Laboratory, Department of Chemistry, Jamia Millia Islamia, Jamia Nagar, New Delhi-110025, India*

[2]*Department of NEUROFARBA, Section of Pharmaceutical and Nutraceutical Sciences, Laboratory of Molecular Modeling, Cheminformatics & QSAR, University of Florence, Polo Scientifico, Via U. Schiff 6, 50019 Sesto Fiorentino, Florence, Italy*

* *Corresponding authors:* E-mail address: amir_sumbul@yahoo.co.in (A. Azam).
Tel.: +91-11-26981717/3254; fax: +91 11 26980229
claudiu.supuran@unifi.it (Claudiu T. Supuran).
Tel.: +39-055-4573729/3005

Table of Contents

1. S1.2. Procedure for the Synthesis of Intermediates (**9-16**) S2-S3
2. S1.3. Synthesis Procedure for Carboximidoyl chlorides (**17-24**) S3-S4
3. S1.4. Procedure for synthesis of 4-(piperazin-1-yl)benzene-1-sulfonamide (**26**) S4
4. S1.5. Procedure for synthesis and characterization data of target benzenesulfonamides (**27-34**) S4-S7
5. S2.1. Carbonic anhydrase inhibition assay S7-S8
6. S2.2. Molecular modeling S8-S10
7. ^{1}H NMR and ^{13}C NMR of Compound **27** S11
8. ^{1}H NMR and ^{13}C NMR of Compound **28** S12
9. ^{1}H NMR and ^{13}C NMR of Compound **29** S13
10. ^{1}H NMR and ^{13}C NMR of Compound **30** S14
11. ^{1}H NMR and ^{13}C NMR of Compound **31** S15
12. ^{1}H NMR and ^{13}C NMR of Compound **32** S16
13. ^{1}H NMR and ^{13}C NMR of Compound **33** S17
14. ^{1}H NMR and ^{13}C NMR of Compound **34** S18
15. References S19-S21

S1. Experimental Protocols

S1.1. Materials and Methods

All the required chemicals were purchased from Merck and Aldrich Chemical Company (USA). The reagents were of analytical grade and were used as supplied. Percolated aluminum sheets (Silica gel 60 F_{254}, Merck Germany) were employed for thin-layer chromatography (TLC). The synthesized compounds were visualized on TLC using ultraviolet (UV) light (λ = 254 nm). The melting points of all the compounds were observed on the Veego instrument with model specifications REC-22038 A2 and are uncorrected. ^{1}H NMR and ^{13}C NMR were recorded on a Jeol-500 MHz and Jeol-125 MHz spectrometers, respectively, using DMSO-d_6 as a solvent and trimethylsilane (TMS) as the internal standard. Splitting patterns are designated as follows; s, singlet; d, doublet; t: triplet: m, multiplet; Ar: aromatic. Mass spectra of all the compounds were recorded by ESI-MS (AB-Sciex 2000, Applied Biosystem).

Safety statement

No unexpected or unusually high safety hazards were encountered during experiments of this research work.

S1.2. Procedure for the Synthesis of Arylaldoximes (9-16)

Sodium hydroxide 3N (75.0 mmol) was added drop wise to a stirred suspension of hydroxylamine hydrochloride (75.0 mmol) in 30 mL of water at 0 ºC. To this mixture different arylaldehydes (1-8, 67.5mmol) taken in 40 mL ethanol were added dropwise and the reaction mixture was heated under reflux for 14-20 hours at 90 ºC. The mixture was cooled, poured onto ice cold water to afford the arylaldoximes which were filtered and dried. However some oximes were obtained by the process of extraction which was carried out with

ethyl acetate and water. The organic layer was washed with brine, dried over anhydrous Na_2SO_4, filtered and then concentrated for the next step.

N-[(E)-(pyridin-4-yl)methylidene]hydroxylamine (9): Yield: 84%; molecular formula: $C_6H_6N_2O$; molecular weight: 122.12; ESI-MS (m/z): [M^+ + 1] 123.02

4-[(E) (Hydroxyimino)methyl]phenol (10): Yield: 84%; molecular formula: $C_7H_7NO_2$; molecular weight: 137.13; ESI-MS (m/z): [M^+ + 1] 138.05

N-[(E)-Phenylmethylidene]hydroxylamine (11): Yield: 90%; molecular formula: C_7H_7NO; molecular weight: 121.13; ESI-MS (m/z): [M^+ + 1] 122.02

N-[(E)-(4-Chlorophenyl)methylidene]hydroxylamine (12): Yield: 85%; molecular formula: C_7H_6ClNO; molecular weight: 155.58; ESI-MS (m/z): [M^+ + 1] 156.09

N-[(E)-(4-Methoxyphenyl)methylidene]hydroxylamine (13): Yield: 85%; molecular formula: $C_8H_9NO_2$; molecular weight: 151.16; ESI-MS (m/z): [M^+ + 1] 152.03

N-[(E)-(4-Nitrophenyl)methylidene]hydroxylamine (14): Yield: 84%; molecular formula: $C_7H_6N_2O_3$; molecular weight: 166.13; ESI-MS (m/z): [M^+ + 1] 167.03

N-[(E)-(4-fluorophenyl)methylidene]hydroxylamine (15): Yield: 91%; molecular formula: C_7H_6FNO; molecular weight: 139.12; ESI-MS (m/z): [M^+ + 1] 140.05

N-[(E)-(4-Methylphenyl)methylidene]hydroxylamine (16): Yield: 82%; molecular formula: C_8H_9NO; molecular weight: 135.16; ESI-MS (m/z): [M^+ + 1] 136.03

S1.3. Synthesis Procedure for Carboximidoyl chlorides (17-24)

N-Chlorosuccinimide (NCS) (43.91mmol) taken in DMF (60 ml) was added dropwise over a solution of different synthesized arylaldoximes (43.91mmol) in DMF and heated at 60 ºC for 8-12 hours. The completion of the reaction was monitored by the TLC. The reaction mixture was cooled at room temperature, poured onto ice cold water, and then extracted with tetrabutylmethyether (TBME). The organic layer was filtered and evaporated to dryness (at 30 ºC) to get the desired carboximidoyl chlorides **17-24**.

N-Hydroxypyridine-4-carboximidoyl chloride (17): Yield: 75 %; yellow solid; molecular formula: $C_6H_5ClN_2O$; molecular weight: 156.56; ESI-MS (m/z): [M^+ + 1] 157.01

N,4-dihydroxybenzene-1-carboximidoyl chloride (18): Yield: 75 %; yellow solid; molecular formula: $C_7H_6ClNO_2$; molecular weight: 171.58; ESI-MS (m/z): [M^+ + 1] 172.01

N-Hydroxybenzenecarboximidoyl chloride (19): Yield: 75 %; yellow solid; molecular formula: C_7H_6ClNO; molecular weight: 155.58; ESI-MS (m/z): [M^+ + 1] 156.06

4-Chloro-N-hydroxybenzene-1-carboximidoyl chloride (20): Yield: 87%; white solid; molecular formula: $C_7H_5Cl_2NO$; molecular weight: 190.02; ESI-MS (m/z): [M^+ + 1] 190.01

N-Hydroxy-4-methoxybenzene-1-carboximidoyl chloride (21): Yield: 75%; white solid; molecular formula: $C_8H_8ClNO_{2;}$ molecular weight: 185.60; ESI-MS (m/z): [M^+ + 1] 186.07

N-Hydroxy-4-nitrobenzene-1-carboximidoyl chloride (22): Yield: 86%; white solid; molecular formula: $C_7H_5ClN_2O_{3;}$ molecular weight: 200.57; ESI-MS (m/z): [M^+ + 1] 201.00

4-Fluoro-N-hydroxybenzene-1-carboximidoyl chloride (23): Yield: 93%; white solid; molecular formula: C_7H_5ClFNO; molecular weight: 173.57; ESI-MS (m/z): [M^+ + 1] 174.01

N-Hydroxy-4-methylbenzene-1-carboximidoyl chloride (24): Yield: 90%; white solid; molecular formula: C_8H_8ClNO; molecular weight: 169.60; ESI-MS (m/z): [M^+ + 1] 170.05

S1.4. Procedure for synthesis of 4-(piperazin-1-yl)benzene-1-sulfonamide (26)

A mixture of 4-fluorobenzenesulfonamide (25, 5 mmol, 1 eqv) and piperazine (20 mmol, 4 eqv) in water (30 mL) was heated at 100 °C overnight. The solid was then filtered, washed with water and toluene, and dried under reduced pressure to give the pure product (**26**).

4-(piperazin-1-yl)benzene-1-sulfonamide (26): Yield: 90%; white solid; molecular formula: $C_{10}H_{15}N_3O_2S$; molecular weight: 241.13; ESI-MS (m/z): [M^+ + 1] 242.09

S1.5. Procedure for synthesis of target benzenesulfonamides (27-34):

The synthesized 4-(piperazin-1-yl)benzene-1-sulfonamide **26** (1 eqv) was stirred in 50% THF: H_2O with sodium carbonate (1eqv) for 30 min. The different substituted carboximidoyl

chlorides **17-24** (1eqv) dissolved in THF was added drop wise to the above solution. Progress of the reaction was monitored by TLC visualized under UV light (λ = 254 nm). The reaction was completed in 24-48 h at room temperature. The reaction mixture was poured onto ice cold water, and the solid product obtained was filtered, dried and recrystallized from ethanol and dichloromethane to get the title benzenesulfonamides **27-34**.

4-{4-[(Hydroxyimino)(pyridin-4-yl)methyl]piperazin-1-yl}benzene-1-sulfonamide (27): Yield: 90%; white solid; m.p: 310-311 °C; ^{1}H NMR (500 MHz, DMSO-d_6): δ= 10.54 (s, 1H, -OH), 8.60 (d, 2H, J = 6.2 Hz, -Ar-H), 7.64 (d, 2H, J = 8.9 Hz, -Ar-H), 7.50-7.49 (m, 2H, -Ar-H), 7.08-7.03 (m, 4H, Ar–H+NH2), 3.44-3.42 (m, 2H, -ppz), 3.35-3.34 (m, 4H, -ppz), 1.06-1.03 (m, 2H, -ppz); ^{13}C NMR (125 MHz, DMSO-d_6): δ= 152.98, 150.42, 149.86, 142.41, 133.05, 127.14, 122.89, 114.02, 56.07, 47.89, 47.73, 18.59. ESI-MS (m/z): $[M+H]^+$ 362.30. Anal. Calcd. For $C_{16}H_{19}N_5O_3S$: C, 53.17; H, 5.30; N, 19.38; S, 8.87. Found: C, 52.87; H, 5.10; N, 19.18; S, 8.63.

4-{4-[(Hydroxyimino)(4-hydroxyphenyl)methyl]piperazin-1-yl}benzene-1-sulfonamide (28): Yield: 65%; white solid; m.p: 320-321 °C; ^{1}H NMR (500 MHz, DMSO-d_6): δ= 9.42 (s, 1H, -N-OH), 8.72 (s, 1H, -OH), 7.61 (d, 2H, J = 8.9 Hz, -Ar-H), 7.26-7.21 (m, 4H, -Ar-H), 7.08 (s, 2H, NH_2), 7.00 (d, 2H, J = 8.9 Hz, -Ar-H), 3.25-3.23 (m, 4H, -ppz), 3.00-3.98 (m, 4H, -ppz), ^{13}C NMR (125 MHz, DMSO-d_6): δ=163.41, 158.82, 153.47, 139.20, 133.20, 129.47, 129.39, 128.48, 127.71, 114.61, 55.22, 47.34, 47.27, ESI-MS (m/z): $[M+H]^+$ 377.10. Anal. Calcd. For $C_{17}H_{20}N_4O_4S$: C, 54.24; H, 5.36; N, 14.88; S, 8.52. Found: C, 54.23; H, 5.12; N, 14.73; S, 8.44.

4-{4-[(Hydroxyimino)(phenyl)methyl]piperazin-1-yl}benzene-1-sulfonamide (29): Yield: 88%; white solid; m.p: 297-298 °C; ^{1}H NMR (500 MHz, DMSO-d_6): δ= 9.49 (s, 1H, -OH), 7.63 (d, 2H, J = 8.9 Hz, -Ar-H), 7.44-7.39 (m, 5H, -Ar-H), 7.08 (s, 2H, NH_2), 7.03 (d, 2H, J = 8.9 Hz, -Ar-H), 3.28 (m, 4H, -ppz), 3.03 (m, 4H, -ppz); ^{13}C NMR (125 MHz, DMSO-d_6):

δ=157.87, 152.93, 149.08, 133.16, 131.17, 129.08, 128.29, 127.18, 114.08, 46.91, 46.85; ESI-MS (m/z): $[M+H]^+$ 361.13. Anal. Calcd. For $C_{17}H_{20}N_4O_3S$: C, 56.65; H, 5.59; N, 15.54; S, 8.90. Found: C, 56.61; H, 5.39; N, 15.45; S, 8.80.

4-{4-[(4-Chlorophenyl)(hydroxyimino)methyl]piperazin-1-yl}benzene-1-sulfonamide (30): Yield: 85%; white solid; m.p: 321 °C; ^{1}H NMR (500 MHz, DMSO-d_6): δ= 9.65 (s, 1H, -OH), 7.64 (d, 2H, J = 8.9 Hz, -Ar-H), 7.52-7.50 (m, 2H, -Ar-H), 7.45-7.43 (m, 2H, -Ar-H), 7.09 (s, 2H, NH_2), 7.04-7.02 (m, 2H, -Ar-H), 3.36-3.28 (m, 4H, -ppz), 3.04-3.02 (m, 4H, -ppz); ^{13}C NMR (125 MHz, DMSO-d_6): δ=162.33, 156.67, 152.81, 133.61, 133.19, 131.02, 130.23, 129.76, 128.33, 127.10, 114.01, 56.05, 47.90, 47.76, 46.90, 46.78, 18.59; ESI-MS (m/z): ESI-MS (m/z): $[M+H]^+$ 395.09. Anal. Calcd. For $C_{17}H_{19}ClN_4O_3S$: C, 51.71; H, 4.85; N, 14.19; S, 8.12. Found: C, 51.61; H, 4.70; N, 13.99; S, 8.06.

4-{4-[(Hydroxyimino)(4-methoxyphenyl)methyl]piperazin-1-yl}benzene-1-sulfonamide (31): Yield: 75%; white solid; m.p: 310-311 °C; ^{1}H NMR (500 MHz, DMSO-d_6): δ= 9.64 (s, 1H, OH), 7.63 (d, 2H, J = 8.9 Hz, -Ar-H), 7.48 (d, 1H, J = 2.05 Hz, -Ar-H), 7.40-7.38 (m, 1H, -Ar-H), 7.22 (d, 1H, J = 8.9 Hz, -Ar-H), 7.08 (s, 2H, NH_2), 7.04 (d, 2H, J = 9.6 Hz, -Ar-H), 6.99 (d, 1H, J = 8.9 Hz, -Ar-H), 3.89 (s, 3H, CH_3), 3.32-3.29 (m, 4H, -ppz), 3.03-3.02 (m, 4H, -ppz); ^{13}C NMR (125 MHz, DMSO-d_6): δ=162.32, 159.55, 157.32, 156.13, 154.84, 152.83, 133.15, 130.64, 130.61, 129.30, 127.09, 123.74, 122.87, 120.59, 113.98, 113.51, 112.43, 56.20, 55.15, 47.07, 46.79, 35.79, 30.78; ESI-MS (m/z): $[M+H]^+$ 391.10. Anal. Calcd. For $C_{18}H_{22}N_4O_4S$: C, 55.37; H, 5.68; N, 14.35; S, 8.21. Found: C, 55.28; H, 5.39; N, 14.10; S, 8.50.

4-{4-[(Hydroxyimino)(4-nitrophenyl)methyl]piperazin-1-yl}benzene-1-sulfonamide (32):

Yield: 91%; Yellowish solid; m.p: 302-303 °C; ^{1}H NMR (500 MHz, DMSO-d_6): δ= 9.87 (s, 1H, -OH), 8.30 (d, 2H, J = 8.9 Hz, -Ar-H), 7.70 (d, 2H, J = 8.9 Hz, -Ar-H), 7.63 (d, 2H, J =

8.9 Hz, -Ar-H), 7.09 (s, 2H, NH_2), 7.05 (d, 2H, J = 8.9 Hz, -Ar-H), 3.32-3.30 (m, 4H, -ppz), 3.05-3.04 (m, 4H, -ppz); ^{13}C NMR (125 MHz, DMSO-d_6): δ=162.38, 156.19, 152.80, 147.56, 137.73, 133.26, 130.64, 127.13, 123.44, 114.08, 56.08, 46.84, 46.75, ESI-MS (m/z): $[M+H]^+$ 405.12. Anal. Calcd. For $C_{17}H_{19}N_5O_5S$: C, 50.36; H, 4.72; N, 17.27; S, 7.91. Found: C, 50.65; H, 4.97; N, 16.98; S, 7.89.

4-{4-[(4-Fluorophenyl)(hydroxyimino)methyl]piperazin-1-yl}benzene-1-sulfonamide (33): Yield: 90%; white solid; m.p: 263 °C; ^1H NMR (500 MHz, DMSO-d_6): δ= 9.49 (s, 1H, -OH), 7.59 (d, 2H, J = 8.9 Hz, -Ar-H), 7.44 (d, 2H, J = 8.9 Hz, -Ar-H), 7.33 (d, 2H, J = 8.2 Hz, -Ar-H), 7.03 (s, 2H, NH_2), 6.96 (d, 2H, J = 8.9 Hz, -Ar-H), 3.20 (s, 4H, -ppz), 2.98 (s, 4H, -ppz); ^{13}C NMR (125 MHz, DMSO-d_6): δ=159.57, 154.16, 135.20, 133.01, 131.84, 130.50, 129.60, 128.34, 115.41, 47.74, 47.54; ESI-MS (m/z): $[M+H]^+$ 379.10. Anal. Calcd. For $C_{17}H_{19}FN_4O_3S$: C, 53.96; H, 5.06; N, 14.81; S, 8.47. Found: C, 53.70; H, 5.25; N, 15.04; S, 8.21.

4-{4-[(Hydroxyimino)(4-methylphenyl)methyl]piperazin-1-yl}benzene-1-sulfonamide (34): Yield: 85%; white solid; m.p: 263-264 °C; ^1H NMR (500 MHz, DMSO-d_6): δ= 9.45 (s, 1H, -OH), 7.63 (d, 2H, J = 8.9 Hz, -Ar-H), 7.32 (d, 2H, J = 7.5 Hz, -Ar-H), 7.25 (d, 2H, J = 8.2 Hz, -Ar-H), 7.08 (s, 2H, NH_2), 7.04 (d, 2H, J = 8.9 Hz, -Ar-H), 3.29-3.27 (m, 4H, -ppz), 3.03-3.01 (m, 4H, -ppz), 2.33 (s, 3H, CH_3); ^{13}C NMR (125 MHz, DMSO-d_6): δ=162.32, 157.66, 152.85, 138.39, 133.13, 129.00, 128.73, 128.07, 127.09, 113.96, 54.93, 46.93, 46.81, 20.95; ESI-MS (m/z): $[M+H]^+$ 375.20. Anal. Calcd. For $C_{18}H_{22}N_4O_3S$: C, 57.73; H, 5.92; N, 14.96; S, 8.56. Found: C, 57.71; H, 5.83; N, 14.83; S, 8.51.

S2. Pharmacological evaluation

S2.1. Carbonic anhydrase inhibition assay

An Applied Photophysics stopped-flow instrument has been used for assaying the CA catalyzed CO_2 hydration activity[1]. Phenol red (at a concentration of 0.2 mM) has been used

as indicator, working at the absorbance maximum of 557 nm, with 20 mM Hepes (pH 7.5) as buffer, and 20 mM Na_2SO_4 (for maintaining constant the ionic strength), following the initial rates of the CA-catalyzed CO2 hydration reaction for a period of 10−100 s.15 The CO_2 concentrations ranged from 1.7 to 17 mM for the determination of the kinetic parameters and inhibition constants. For each inhibitor at least six traces of the initial 5−10% of the reaction have been used for determining the initial velocity. The uncatalyzed rates were determined in the same manner and subtracted from the total observed rates. Stock solutions of inhibitor (0.1 mM) were prepared in distilled−deionized water and dilutions up to 0.01 nM were done thereafter with the assay buffer. Inhibitor and enzyme solutions were preincubated together for 15 min at room temperature prior to assay, in order to allow for the formation of the E−I complex. The inhibition constants were subsequently obtained by nonlinear least-squares methods using PRISM 3 and the Cheng−Prusoff equation, as reported earlier, and represent the mean from at least three different determinations. All CA isofoms were recombinant ones obtained in-house as reported earlier[2,3].

S2.2. Molecular modeling

The crystal structures of CA I (PDB:2NMX)[4], CA II (PDB:3K34)[5], CA IX (PDB:5FL4)[6] and CA XII (PDB:1JD0)[7] used for computational studies were downloaded by Protein Data Bank [8] and prepared according to the Protein Preparation module in Maestro Schrödinger suite, assigning bond orders, adding hydrogens, deleting water molecules, and optimizing H-bonding networks. Finally, energy minimization with a Root Means Square Deviation (RMSD) value of 0.30 was applied using an Optimized Potential for Liquid Simulation (OPLS4) force field [9–13]. Grids for docking were centered in the centroid of the complexed ligand. Docking studies were carried out with the program Glide [9f] using the standard precision (SP) mode. 3D ligand structures were prepared by Maestro [9a]. Molecular dynamics (MD) simulations were performed using Desmond Molecular Dynamics System

(v.7.0) [9g] and OPLS4 force field. For simulations in the CA active sites, the partial charge of the zinc ion was set to 0.8 and a zero bound order was considered between the ligands coordinating atom and the metal[14].All systems were solvated in an orthorhombic box using simple point charge water molecules extended 15 Å away from any protein atom. The system was neutralized with 0.15 M Cl^- and Na^+ ions. The simulation protocol included a starting relaxation step followed by a final production phase of 100 ns. In particular, the relaxation step comprised the following: (a) a stage of 100 ps at 10 K retaining the harmonic restraints on the solute heavy atoms (force constant of 50 Kcal/mol/Å^2) using the NPT ensemble with Brownian dynamics; (b) a stage of 12 ps at 10 K with harmonic restraints on the solute heavy atoms (force constant of 50 Kcal/mol/Å^2), using the NVT ensemble and Berendsen thermostat; (c) a stage of 12 ps at 10 K and 1 atm, retaining the harmonic restraints and using the NPT ensemble and Berendsen thermostat and barostat; (f) a stage of 12 ps at 300 K and 1 atm, retaining the harmonic restraints and using the NPT ensemble and Berendsen thermostat and barostat; (g) a final 24 ps stage at 300 K and 1 atm without harmonic restraints, using the NPT Berendsen thermostat and barostat. The final production phase of MD was run using a canonical NPT Berendsen ensemble at 300 K. During the MD simulation, a time step of 2 fs was used while constraining the bond lengths of H atoms with the M-SHAKE algorithm. The atomic coordinates of the system were saved every 100 ps along the MD trajectory. Protein RMSD, ligand RMSD/RMSF (Root Mean Square Fluctuation) ligand torsions evolution and occurrence of intermolecular H-bonds and hydrophobic contacts were provided by the Simulation Interaction Diagram (SID) implemented in Maestro along with the production phase of the MD simulation. The tool reads the MD trajectory file and identifies ligand/target interactions repeatedly occurring during the simulation time (for instance, a 60% value suggests that the interaction is maintained for 60% of the MD). The 1000 frames resulting

from MDs were clustered using the Conformer Cluster tool implemented in the Schrödinger suite in 10 clusters. Figures were generated with Maestro and Chimera [9][15].

^{1}H NMR of Compound 27

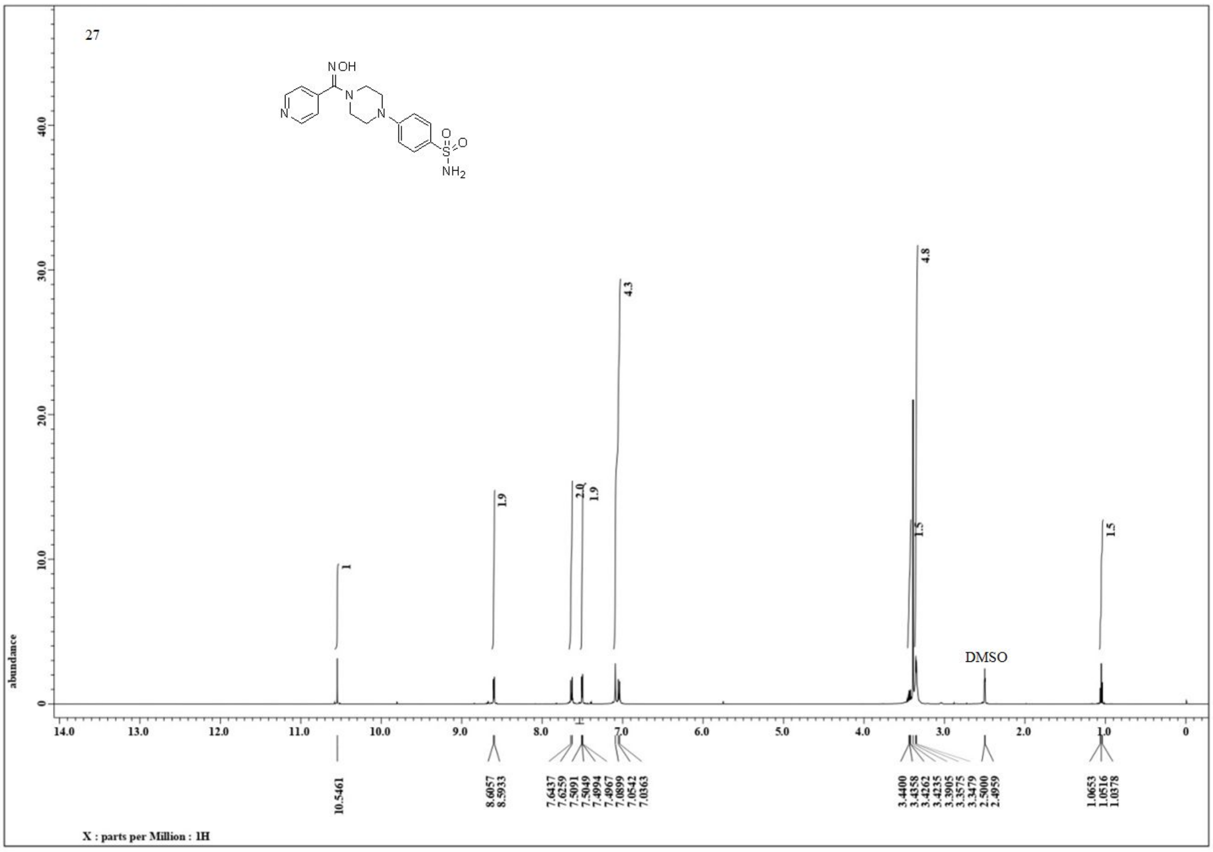

^{13}C NMR of Compound 27

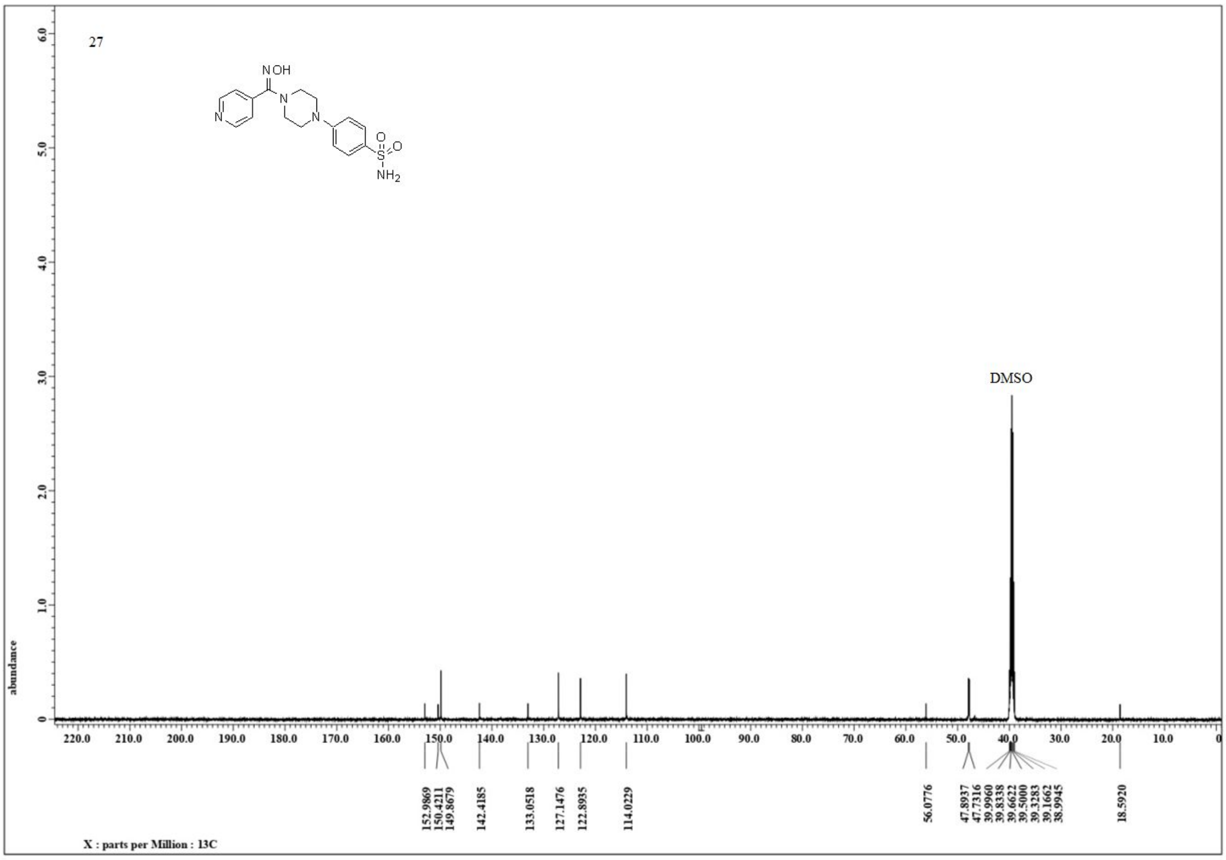

^{1}H NMR of Compound 28

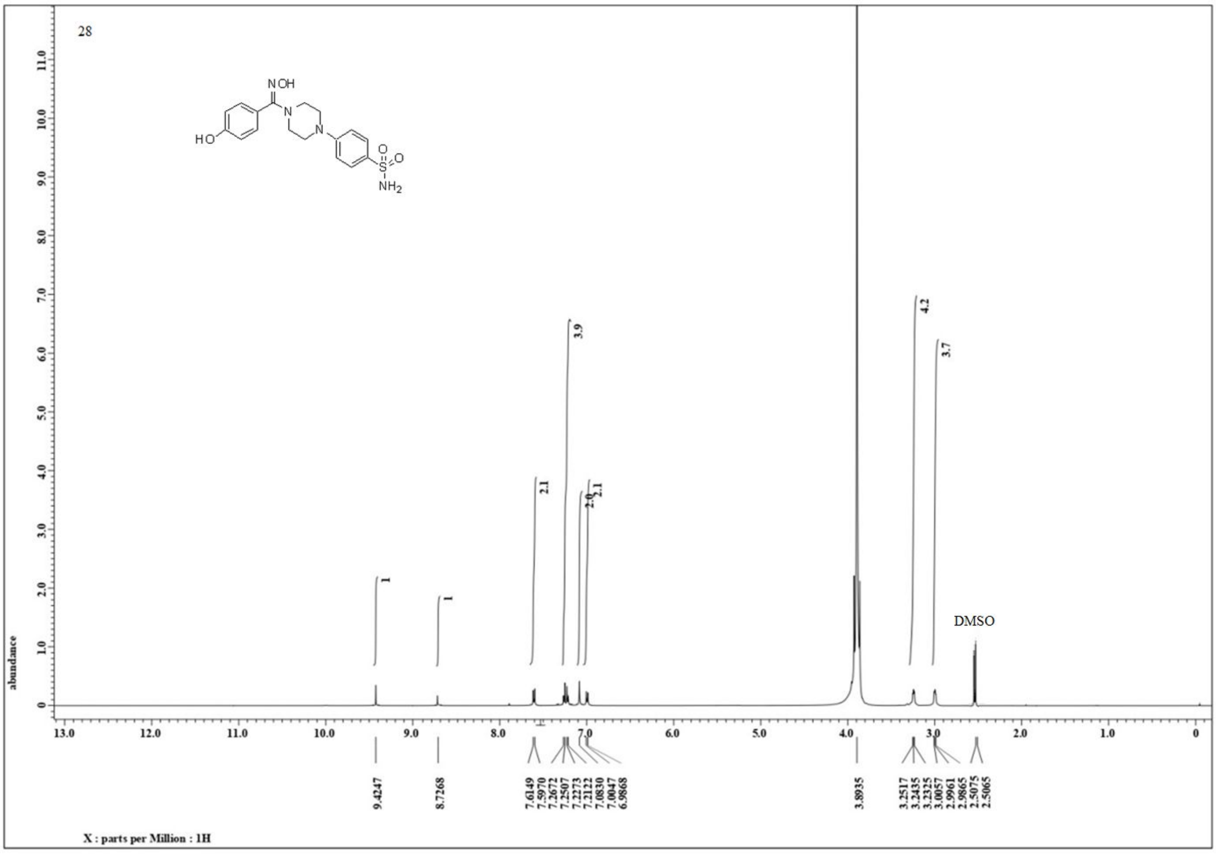

^{13}C NMR of Compound 28

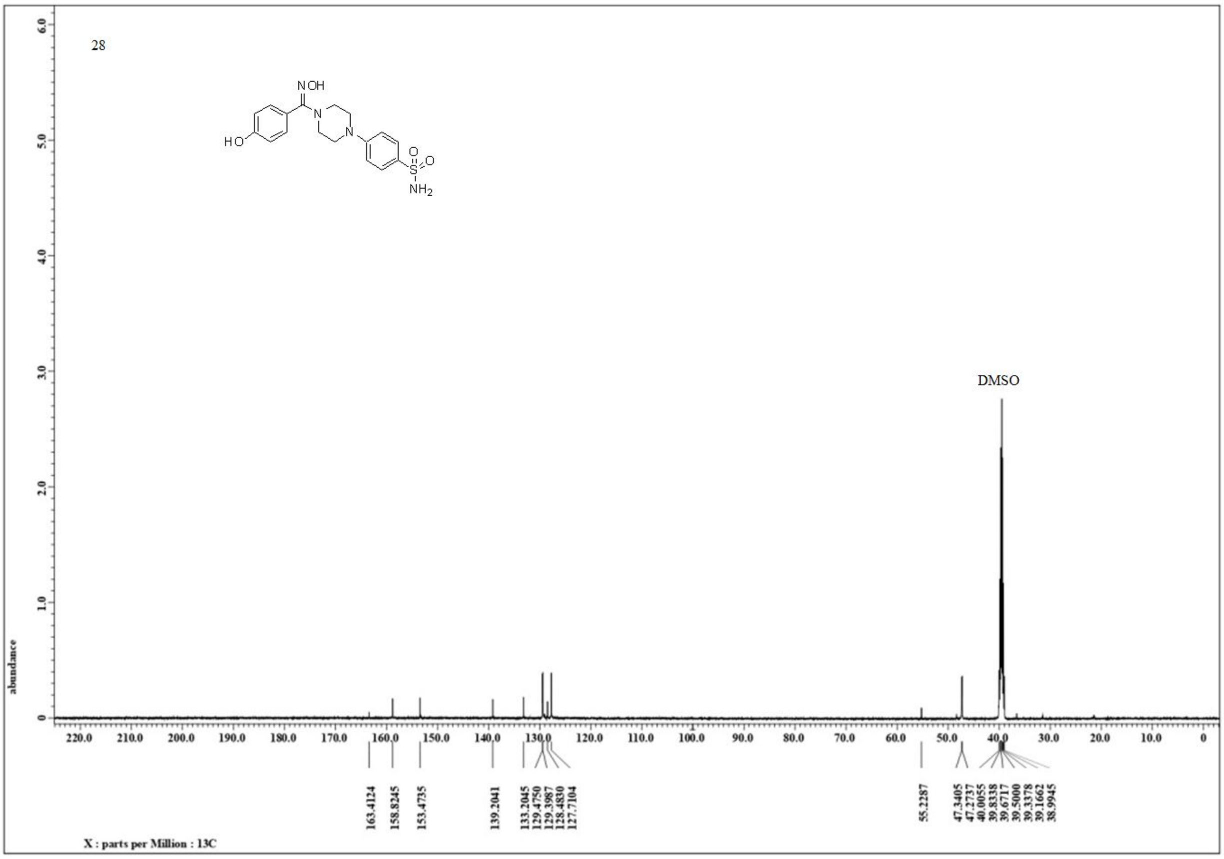

^{1}H NMR of Compound 29

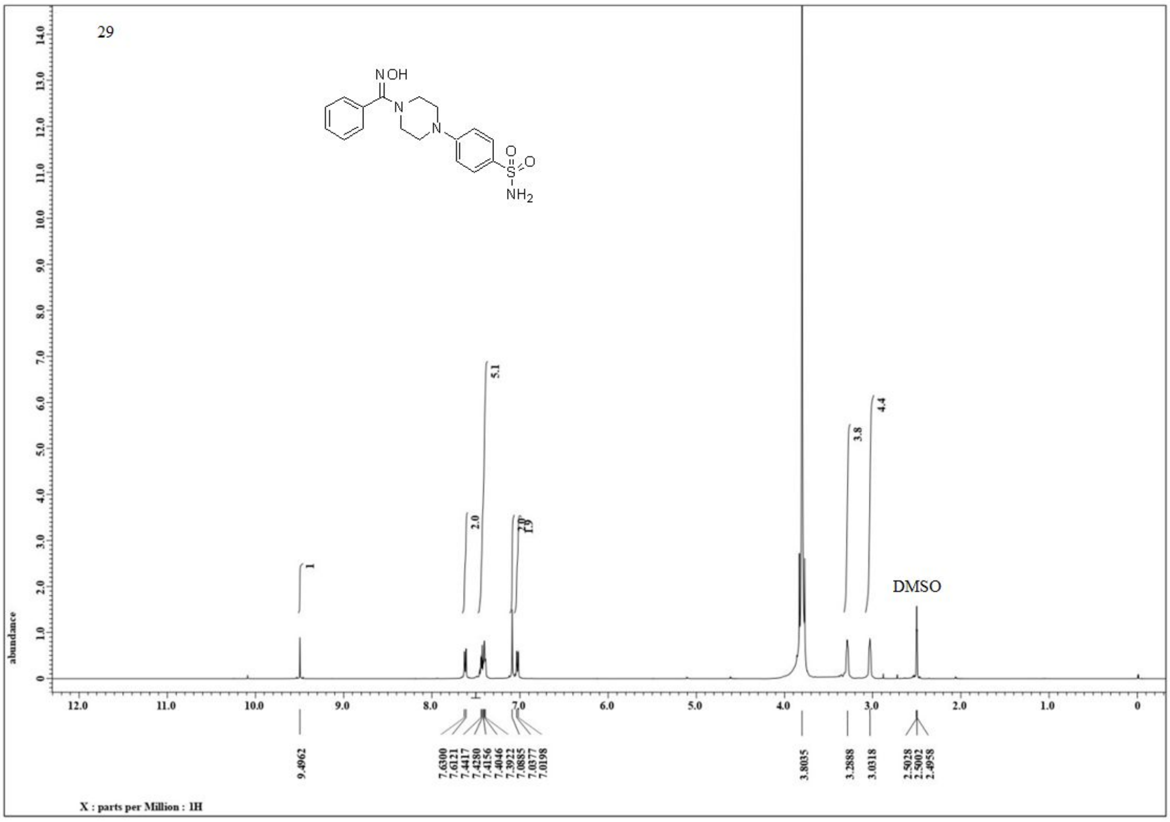

^{13}C NMR of Compound 29

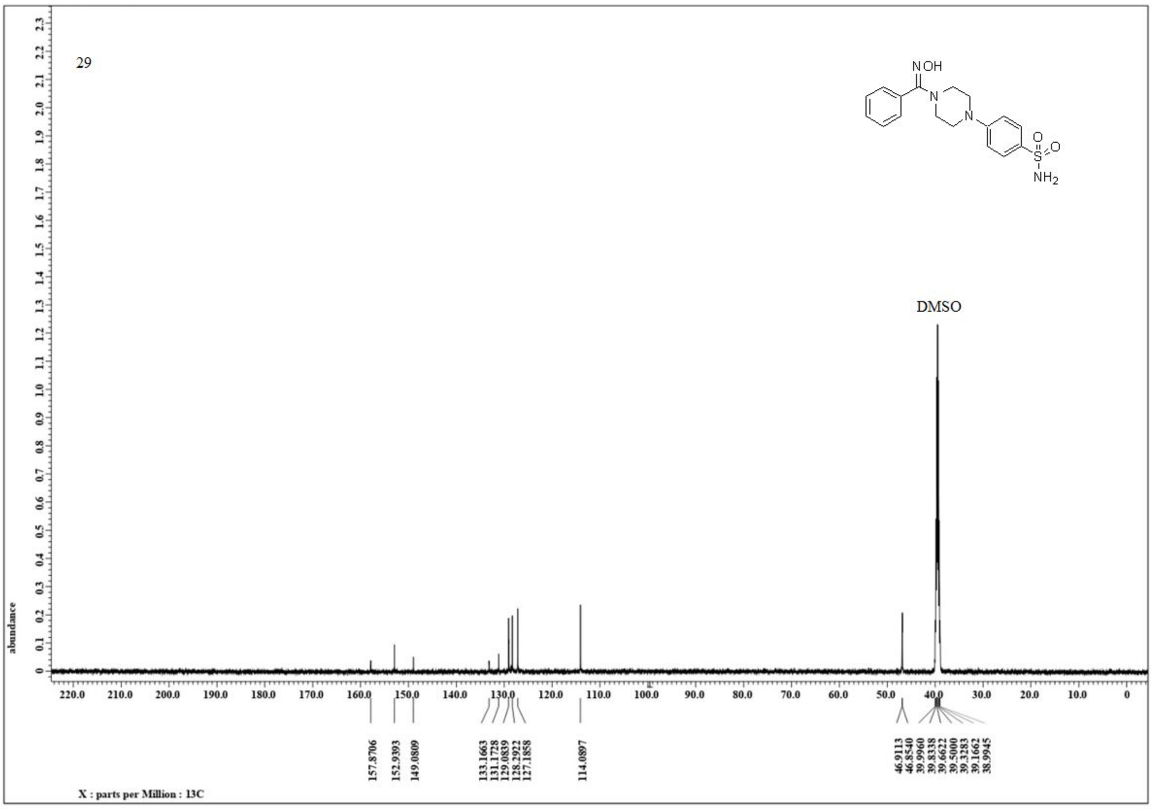

^{1}H NMR of Compound 30

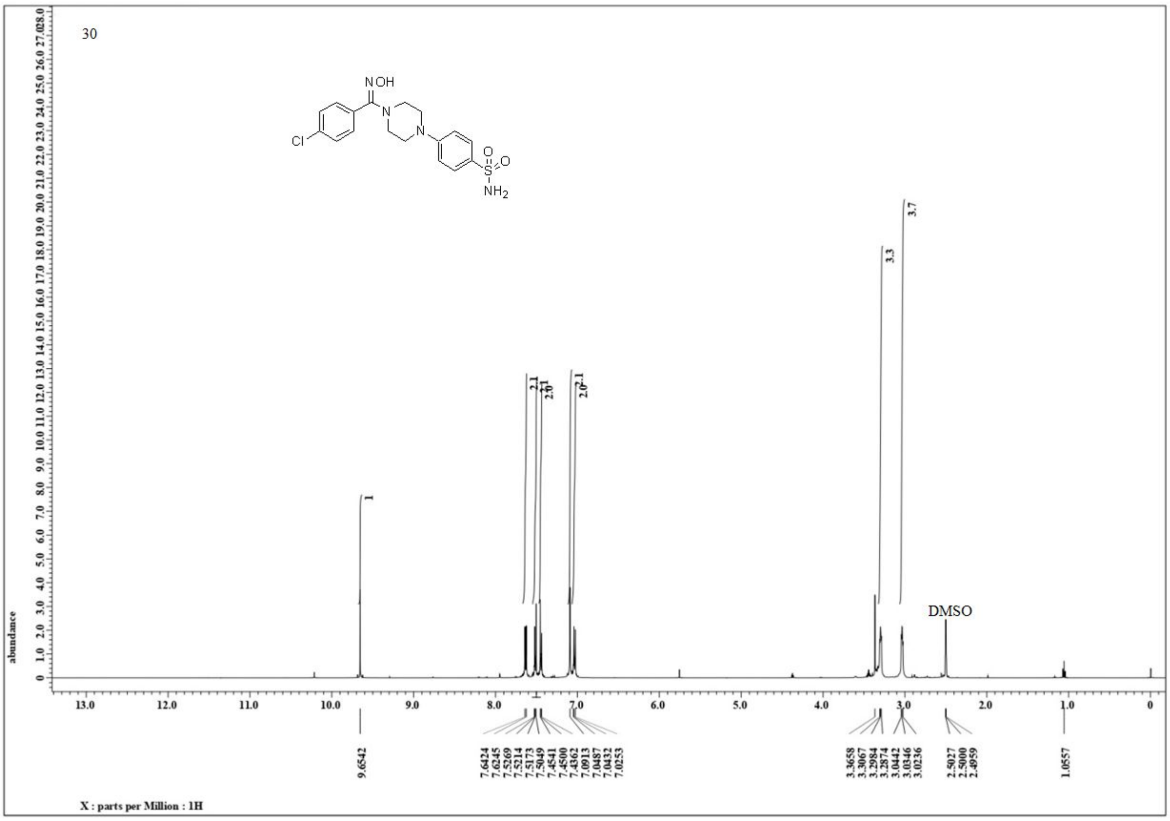

^{13}C NMR of Compound 30

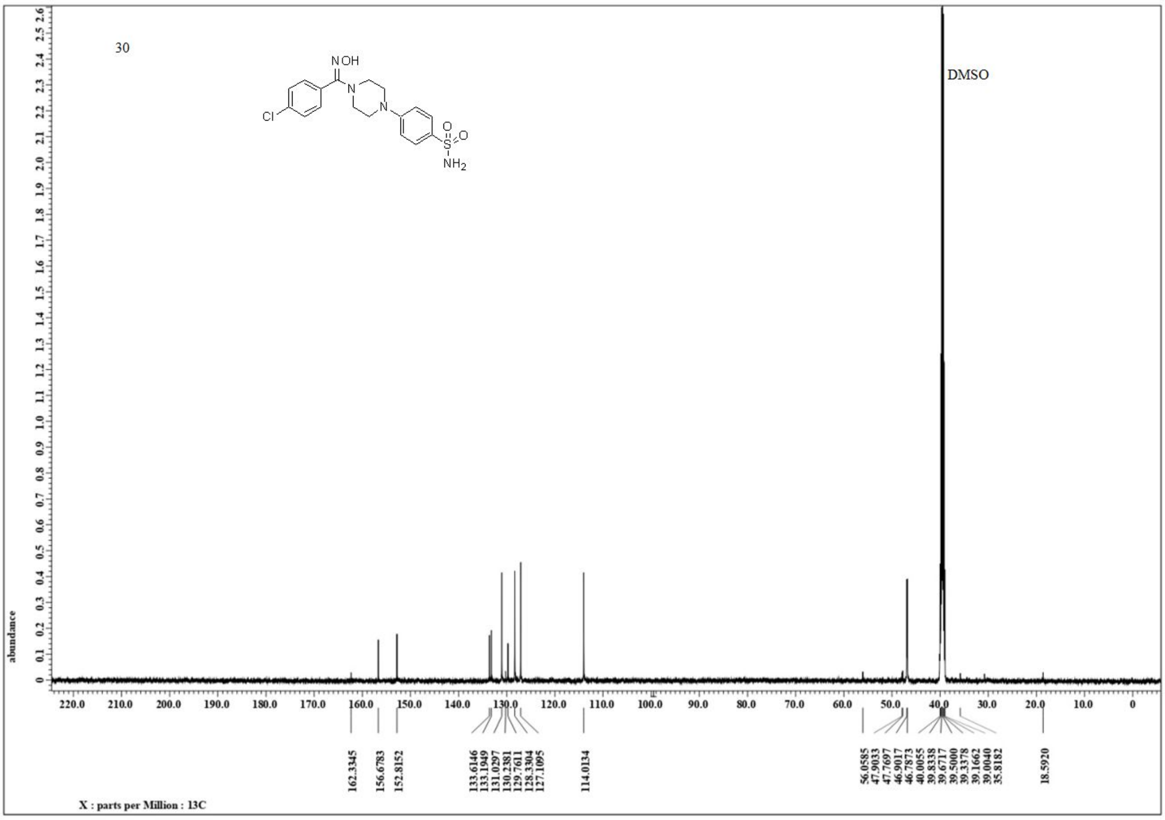

^{1}H NMR of Compound 31

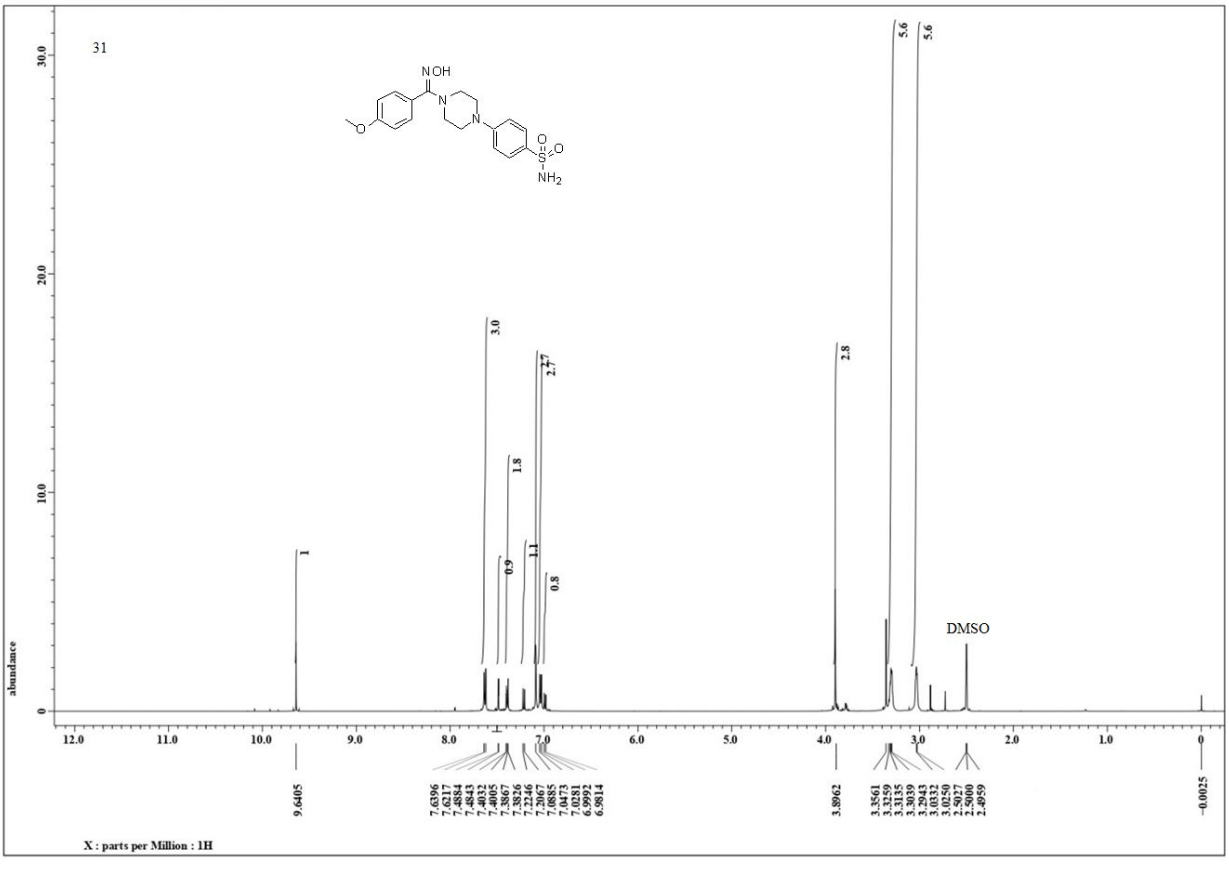

^{13}C NMR of Compound 31

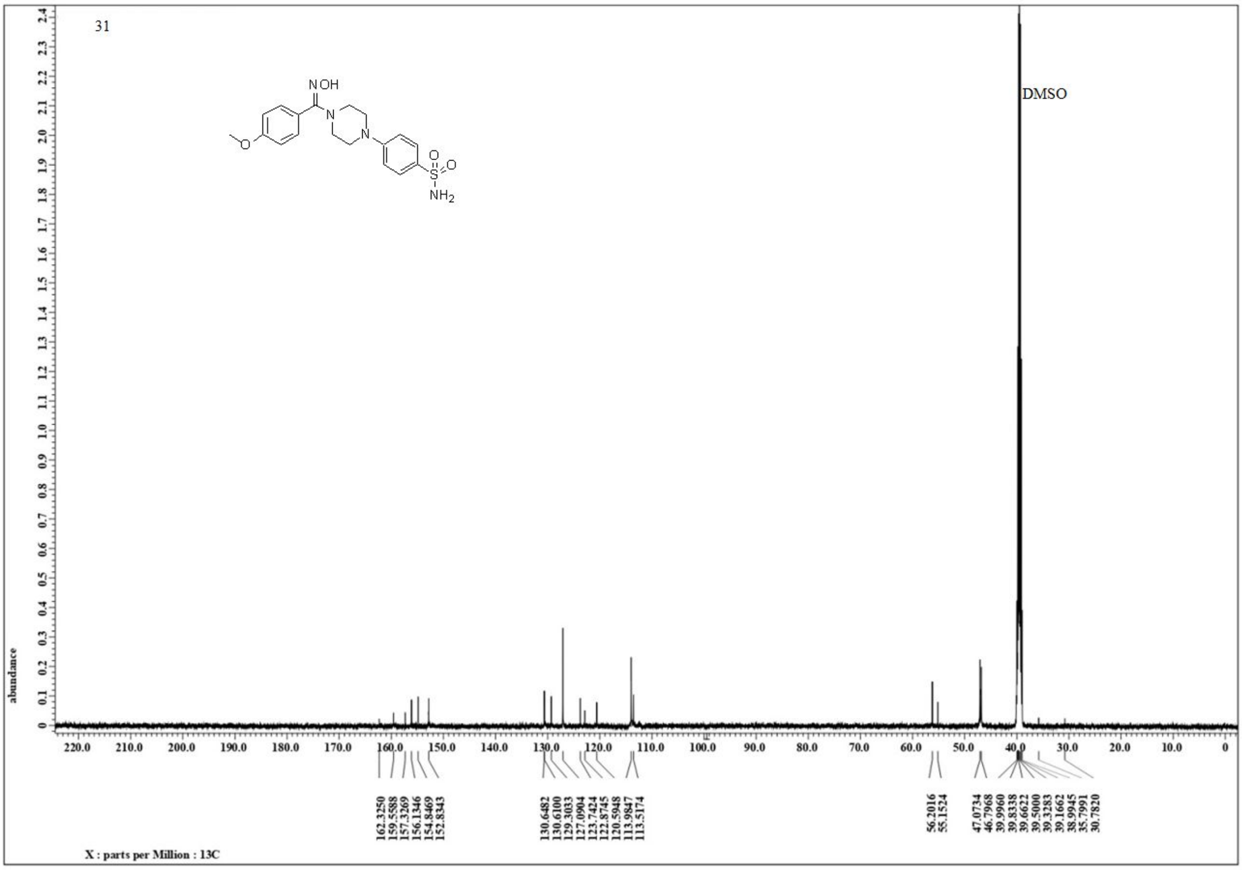

^{1}H NMR of Compound 32

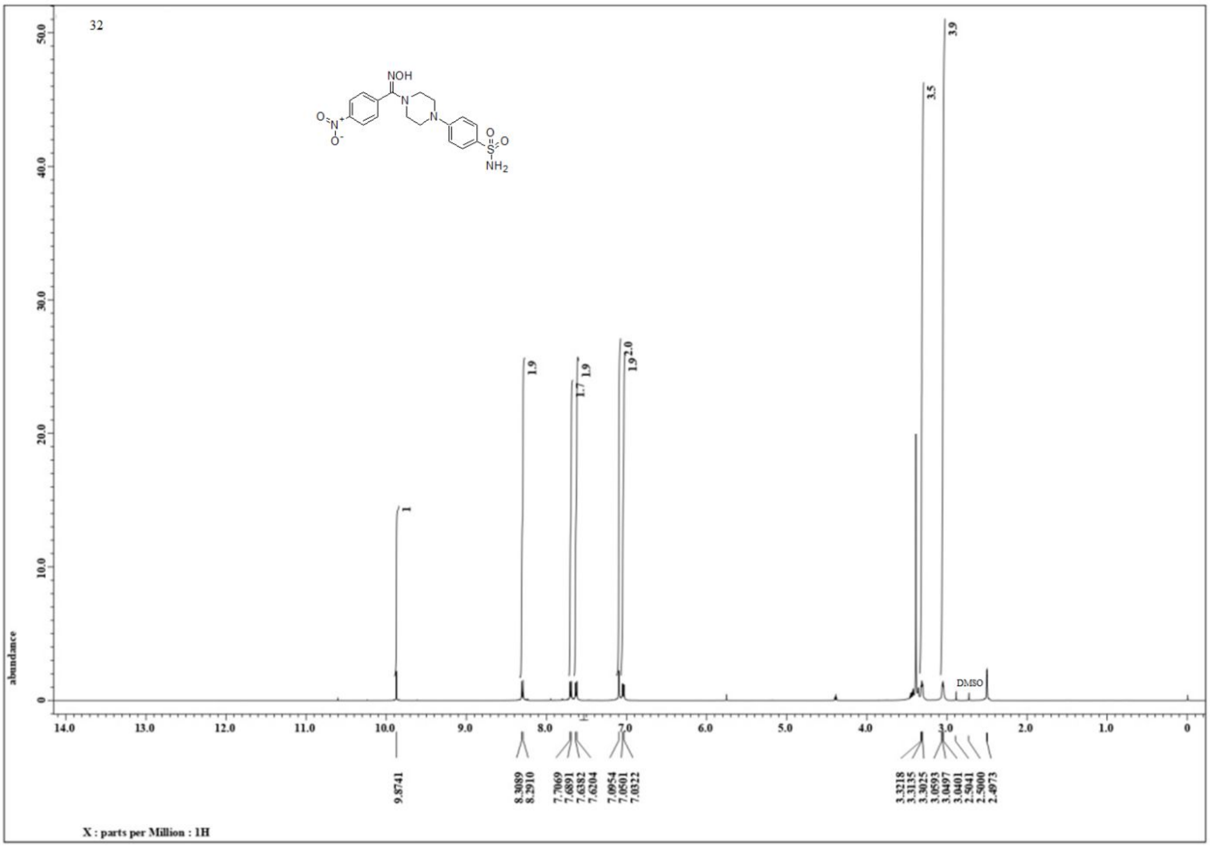

^{13}C NMR of Compound 32

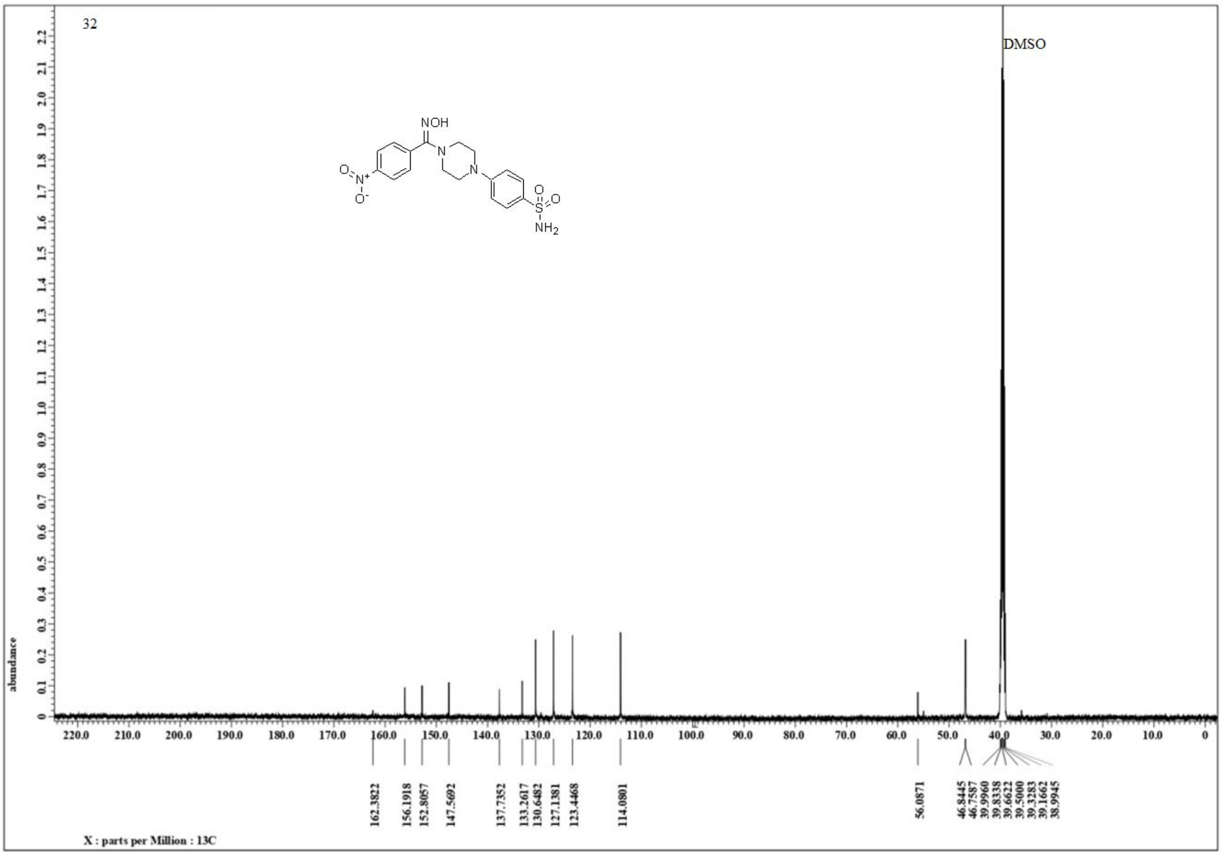

^{1}H NMR of Compound 33

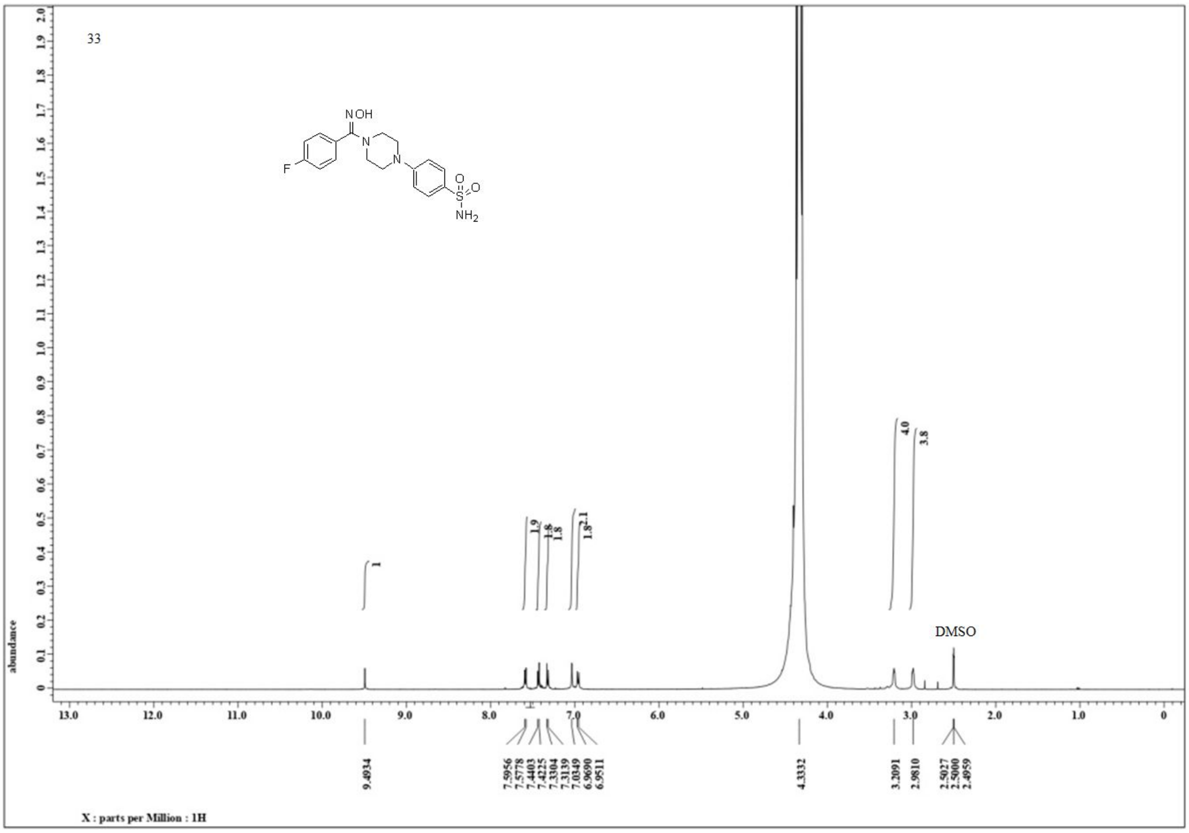

^{13}C NMR of Compound 33

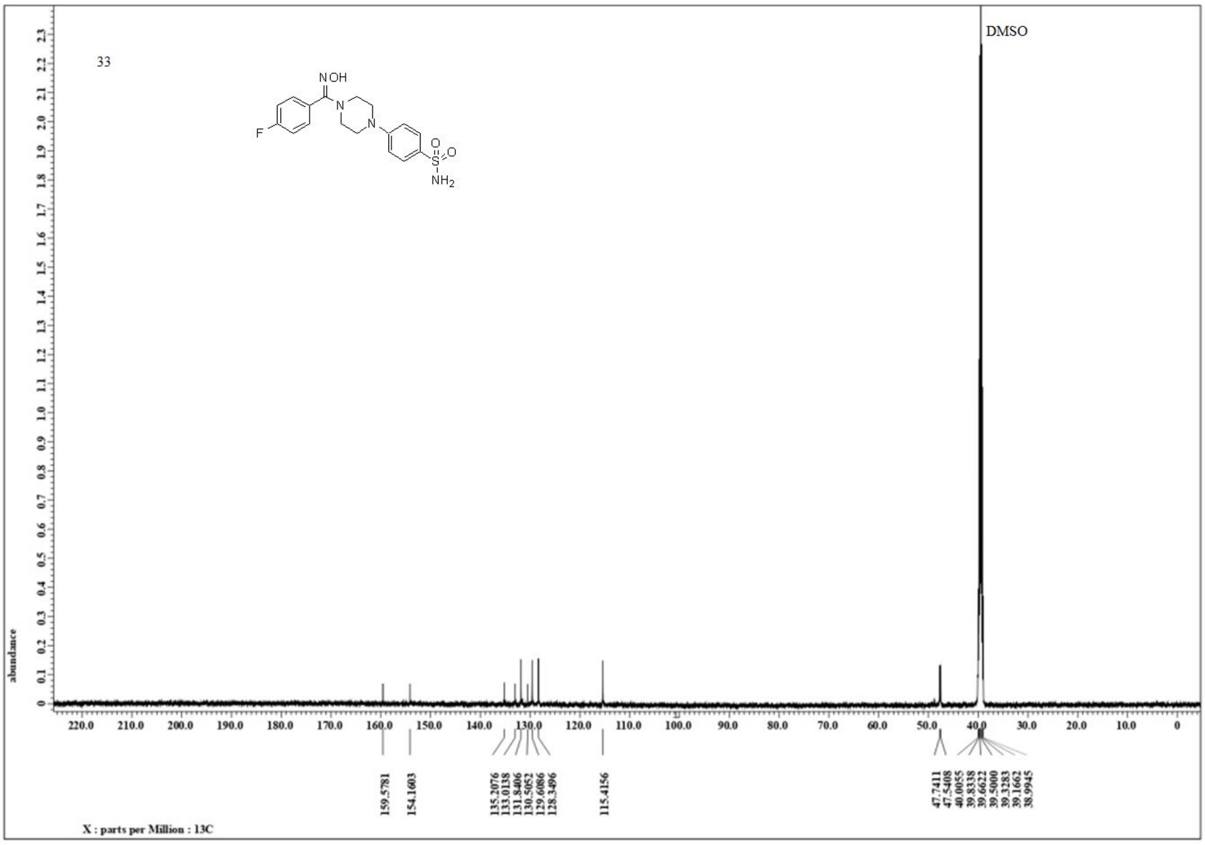

^{1}H NMR of Compound 34

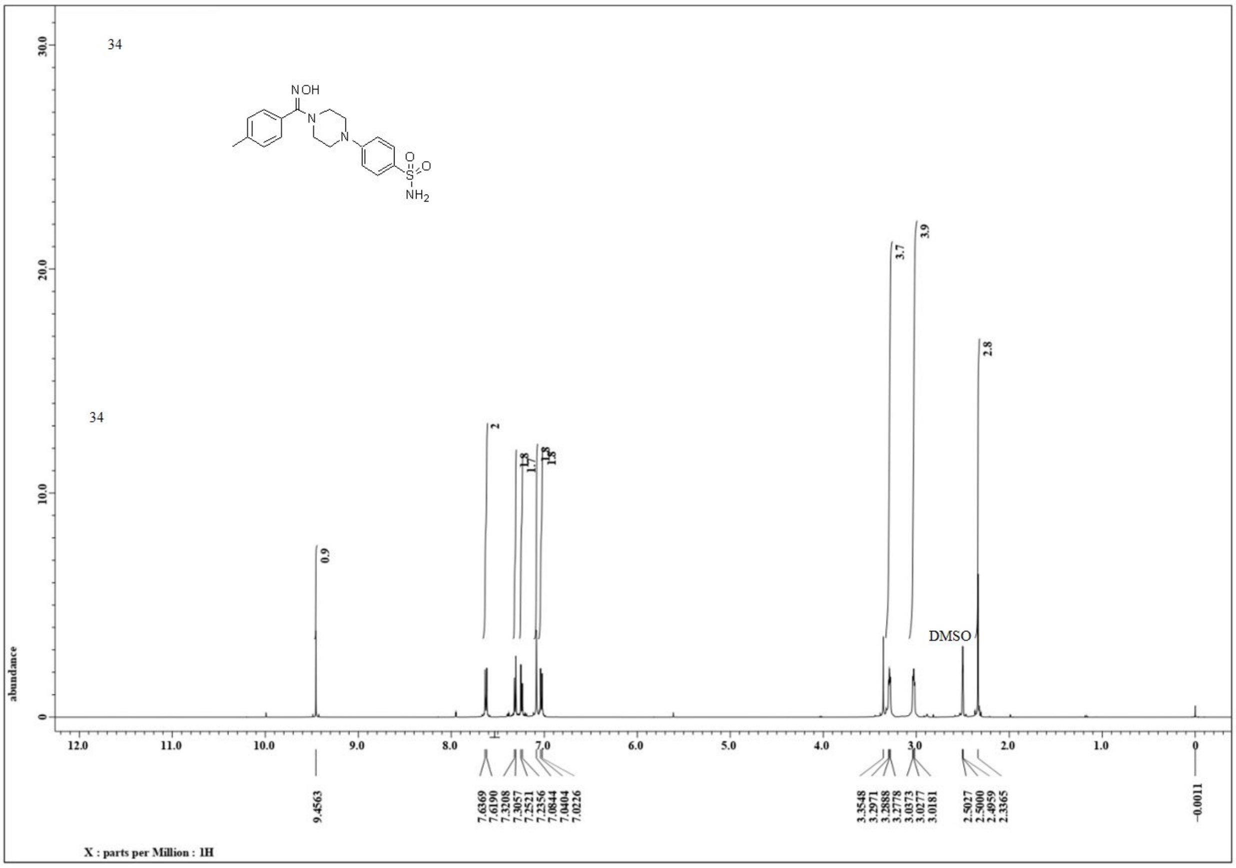

^{13}C NMR of Compound 34

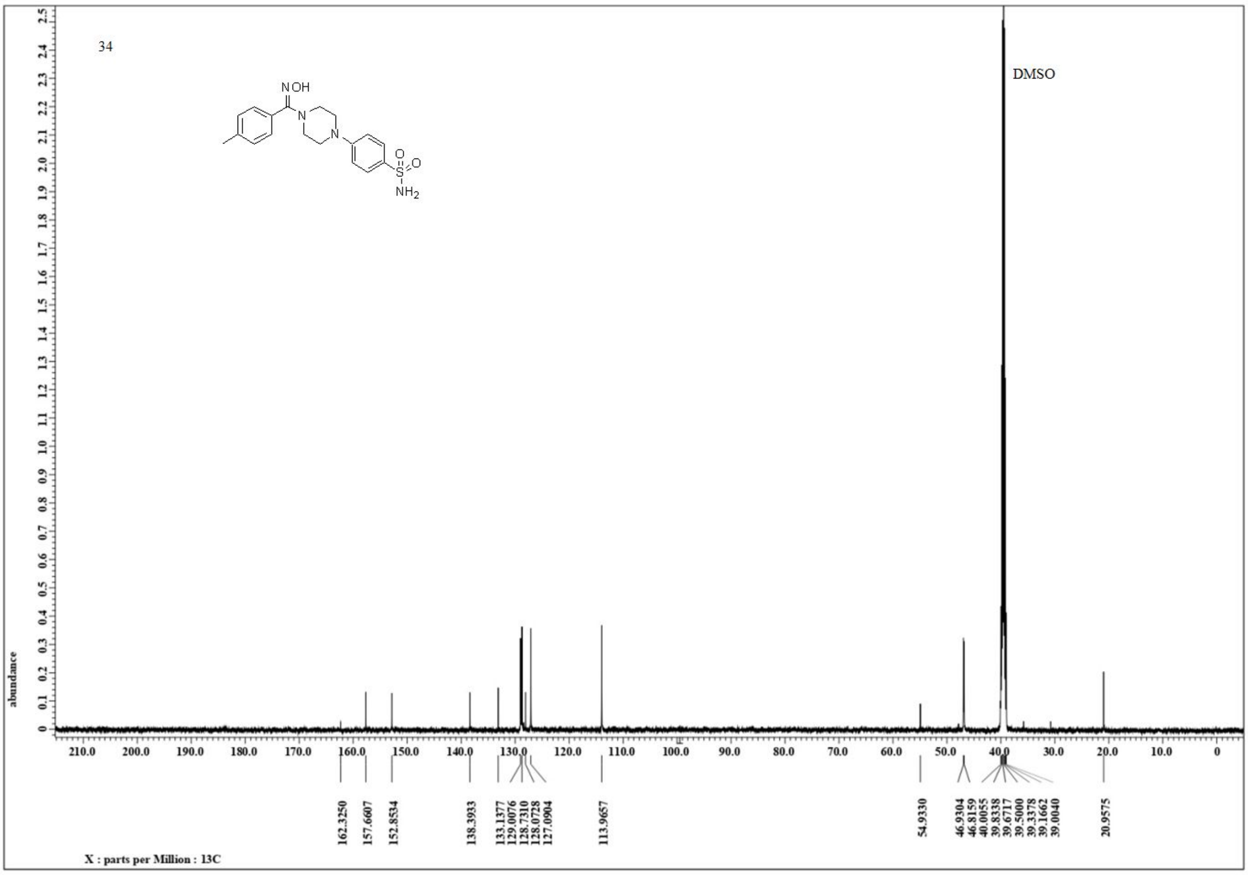

References

(1) Khalifah, R. G. The Carbon Dioxide Hydration Activity of Carbonic Anhydrase. *J. Biol. Chem.* **1971**, *246* (8), 2561–2573.

(2) Wilkinson, B. L.; Bornaghi, L. F.; Houston, T. A.; Innocenti, A.; Supuran, C. T.; Poulsen, S.-A. A Novel Class of Carbonic Anhydrase Inhibitors: Glycoconjugate Benzene Sulfonamides Prepared by "Click-Tailing." *J. Med. Chem.* **2006**, *49* (22), 6539–6548.

(3) Sharonova, T.; Paramonova, P.; Kalinin, S.; Bunev, A.; Gasanov, R. E.; Nocentini, A.; Sharoyko, V.; Tennikova, T. B.; Dar'in, D.; Supuran, C. T.; et al. Insertion of Metal Carbenes into the Anilinic N–H Bond of Unprotected Aminobenzenesulfonamides Delivers Low Nanomolar Inhibitors of Human Carbonic Anhydrase IX and XII Isoforms. *Eur. J. Med. Chem.* **2021**, *218*, 113352.

(4) Srivastava, D. K.; Jude, K. M.; Banerjee, A. L.; Haldar, M.; Manokaran, S.; Kooren, J.; Mallik, S.; Christianson, D. W. Structural Analysis of Charge Discrimination in the Binding of Inhibitors to Human Carbonic Anhydrases I and II. *J. Am. Chem. Soc.* **2007**, *129* (17), 5528–5537.

(5) Behnke, C. A.; Le Trong, I.; Godden, J. W.; Merritt, E. A.; Teller, D. C.; Bajorath, J.; Stenkamp, R. E. Atomic Resolution Studies of Carbonic Anhydrase II. *Acta Crystallogr. Sect. D Biol. Crystallogr.* **2010**, *66* (5), 616–627.

(6) Leitans, J.; Kazaks, A.; Balode, A.; Ivanova, J.; Zalubovskis, R.; Supuran, C. T.; Tars, K. Efficient Expression and Crystallization System of Cancer-Associated Carbonic Anhydrase Isoform IX. *J. Med. Chem.* **2015**, *58* (22), 9004–9009.

(7) Whittington, D. A.; Waheed, A.; Ulmasov, B.; Shah, G. N.; Grubb, J. H.; Sly, W. S.; Christianson, D. W. Crystal Structure of the Dimeric Extracellular Domain of Human Carbonic Anhydrase XII, a Bitopic Membrane Protein Overexpressed in Certain

Cancer Tumor Cells. *Proc. Natl. Acad. Sci.* **2001**, *98* (17), 9545–9550.

(8) Burley, S. K.; Bhikadiya, C.; Bi, C.; Bittrich, S.; Chen, L.; Crichlow, G. V; Christie, C. H.; Dalenberg, K.; Di Costanzo, L.; Duarte, J. M.; et al. RCSB Protein Data Bank: Powerful New Tools for Exploring 3D Structures of Biological Macromolecules for Basic and Applied Research and Education in Fundamental Biology, Biomedicine, Biotechnology, Bioengineering and Energy Sciences. *Nucleic Acids Res.* **2021**, *49* (D1), D437–D451.

(9) Okawa, T.; Aramaki, Y.; Yamamoto, M.; Kobayashi, T.; Fukumoto, S.; Toyoda, Y.; Henta, T.; Hata, A.; Ikeda, S.; Kaneko, M.; et al. Design, Synthesis, and Evaluation of the Highly Selective and Potent G-Protein-Coupled Receptor Kinase 2 (GRK2) Inhibitor for the Potential Treatment of Heart Failure. *J. Med. Chem.* **2017**, *60* (16), 6942–6990.

(10) Lu, C.; Wu, C.; Ghoreishi, D.; Chen, W.; Wang, L.; Damm, W.; Ross, G. A.; Dahlgren, M. K.; Russell, E.; Von Bargen, C. D.; et al. OPLS4: Improving Force Field Accuracy on Challenging Regimes of Chemical Space. *J. Chem. Theory Comput.* **2021**, *17* (7), 4291–4300.

(11) Kaminski, G. A.; Friesner, R. A.; Tirado-Rives, J.; Jorgensen, W. L. Evaluation and Reparametrization of the OPLS-AA Force Field for Proteins via Comparison with Accurate Quantum Chemical Calculations on Peptides. *J. Phys. Chem. B* **2001**, *105* (28), 6474–6487.

(12) Ibrahim, H. S.; Allam, H. A.; Mahmoud, W. R.; Bonardi, A.; Nocentini, A.; Gratteri, P.; Ibrahim, E. S.; Abdel-Aziz, H. A.; Supuran, C. T. Dual-Tail Arylsulfone-Based Benzenesulfonamides Differently Match the Hydrophobic and Hydrophilic Halves of Human Carbonic Anhydrases Active Sites: Selective Inhibitors for the Tumor-Associated HCA IX Isoform. *Eur. J. Med. Chem.* **2018**, *152*, 1–9.

(13) Abo-Ashour, M. F.; Eldehna, W. M.; Nocentini, A.; Ibrahim, H. S.; Bua, S.; Abdel-Aziz, H. A.; Abou-Seri, S. M.; Supuran, C. T. Novel Synthesized SLC-0111 Thiazole and Thiadiazole Analogues: Determination of Their Carbonic Anhydrase Inhibitory Activity and Molecular Modeling Studies. *Bioorg. Chem.* **2019**, *87*, 794–802.

(14) Cau, Y.; Vullo, D.; Mori, M.; Dreassi, E.; Supuran, C.; Botta, M. Potent and Selective Carboxylic Acid Inhibitors of Tumor-Associated Carbonic Anhydrases IX and XII. *Molecules* **2017**, *23* (1), 17.

(15) Pettersen, E. F.; Goddard, T. D.; Huang, C. C.; Couch, G. S.; Greenblatt, D. M.; Meng, E. C.; Ferrin, T. E. UCSF Chimera?A Visualization System for Exploratory Research and Analysis. *J. Comput. Chem.* **2004**, *25* (13), 1605–1612.
